# Bayesian optimization with Gaussian-process-based active machine learning for improvement of geometric accuracy in projection multi-photon 3D printing

**DOI:** 10.1038/s41377-024-01707-8

**Published:** 2025-01-20

**Authors:** Jason E. Johnson, Ishat Raihan Jamil, Liang Pan, Guang Lin, Xianfan Xu

**Affiliations:** 1 School of Mechanical Engineering, Purdue University, 585 Purdue Mall, West Lafayette, IN 47907 USA; 2 Birck Nanotechnology Center, Purdue University, 1205 Mitch Daniels Blvd., West Lafayette, IN 47907 USA; 3 Department of Mathematics, Purdue University, Mathematical Sciences Bldg, 150 N University St, West Lafayette, IN 47907 USA

**Keywords:** Laser material processing, Lithography

## Abstract

Multi-photon polymerization is a well-established, yet actively developing, additive manufacturing technique for 3D printing on the micro/nanoscale. Like all additive manufacturing techniques, determining the process parameters necessary to achieve dimensional accuracy for a structure 3D printed using this method is not always straightforward and can require time-consuming experimentation. In this work, an active machine learning based framework is presented for determining optimal process parameters for the recently developed, high-speed, layer-by-layer continuous projection 3D printing process. The proposed active learning framework uses Bayesian optimization to inform optimal experimentation in order to adaptively collect the most informative data for effective training of a Gaussian-process-regression-based machine learning model. This model then serves as a surrogate for the manufacturing process: predicting optimal process parameters for achieving a target geometry, e.g., the 2D geometry of each printed layer. Three representative 2D shapes at three different scales are used as test cases. In each case, the active learning framework improves the geometric accuracy, with drastic reductions of the errors to within the measurement accuracy in just four iterations of the Bayesian optimization using only a few hundred of total training data. The case studies indicate that the active learning framework developed in this work can be broadly applied to other additive manufacturing processes to increase accuracy with significantly reduced experimental data collection effort for optimization.

## Abstract (web summary)

An active machine learning framework is developed to optimize process parameters in additive manufacturing. Demonstrated for projection multi-photon lithography, it achieves sub-100 nm accuracy in 3D-printed structures with minimal experimentation.

## Graphical abstract

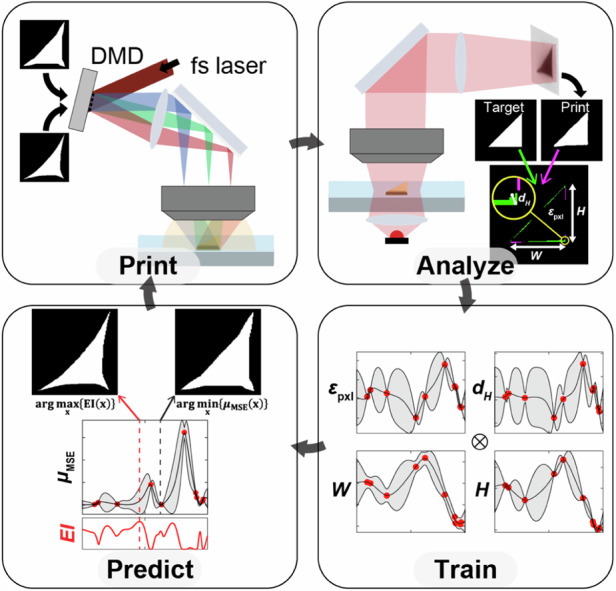

## Introduction

Advancements in additive manufacturing (AM) have transformed the manufacturing industry. The freedom of design, rapid prototyping, and low waste offered by AM, also called 3D printing, have led to its application in a varied range of fields^1^. Yet, its widespread acceptance has been hampered by inconsistency in the quality and accuracy of printed parts. To address this challenge, machine learning (ML) has emerged as a crucial tool. ML has been used to better understand AM through modeling of the underlying processes and to predict 3D printing outcomes to provide actions and parameter sets for achieving optimal results^2^.

One commonly used subset of AM is light-based 3D printing^3^. In these processes, light locally interacts with a material to induce an interaction, changing the material’s properties such that a patterned 3D structure will remain after the process is complete. Two-photon polymerization^4^ is a common light-based AM process for printing nano-scale structures. Two-photon polymerization, also referred to as multi-photon polymerization (MPP), uses high peak intensity laser light with an appropriate wavelength, focused tightly to induce simultaneous absorption of two or more photons to initiate a highly confined polymerization reaction. This nonlinear absorption allows for 3D printing of structures with nanoscale feature sizes and resolution^5^. Although MPP has extraordinary accuracy, the small voxel (polymerization volume) leads to low throughput, limiting its widespread implementation. More recent efforts have focused on improving this throughput of MPP to rival those seen by large-scale stereolithography (SLA) methods^6,7^ by parallelizing the polymerization process. This has been done by simply adding additional focal points^8^, or it has been done similarly to SLA, by using digital light processing and projecting images of high-intensity laser light using a digital micromirror device (DMD) in order to print entire 2D slices of the object at once^9,10^. We have previously presented a projection multi-photon lithography (PMPL) method^10^. This same method will be used for 3D printing in this work.

The quality of structures created by PMPL, like any AM methods using sensitive nonlinear processes, can vary due to numerous parameters and conditions. Therefore, it is beneficial to develop an ML-guided framework for PMPL to reduce the amount of experimentation necessary to determine the optimal parameters for accurate printing. Significant work has been done in applying ML in macro-scale printing methods such as SLA^11–13^, extrusion-based printing^14,15^, and laser metal 3D printing^16^. However, despite the success of ML at the macro-scale, its translation to micro-scale 3D printing has not been well studied. Models, both physical and empirical, have been introduced for a number of phenomena in MPP^17–19^, and many prediction and pre-compensation schemes using such models have been introduced^20,21^. With the rapid advances in ML, it is likely that ML could be leveraged to reduce the necessary size of data and increase the impact of these models and compensation schemes available to MPP. Yet only a few works have presented ML-based methods for micro-scale two-photon polymerization^22–24^. Lee et al. ^24^ presented a convolutional neural network trained with experimentally obtained image datasets for the predicted classification of part quality (cured, uncured, damaged) as a function of laser parameters for the classical serial MPP process. Pingali et al. ^23^ presented a deep neural network for the classification of part quality for the PMPL process. The neural network was trained using a simulated dataset to predict printability as a function of laser parameters and photoresist chemical parameters. These classification methods can provide useful information for determining the feasibility of different areas in the vast 3D printing parameter space. However, these classification methods, when compared to regression-based methods, are limited in their ability to provide quantitative analysis of part quality within the printable class. Yang et al. ^22^ presented an ML regression model for quantification of spatial variation of geometric compliance within the print volume of a Nanoscribe Photonic Professional GT, a commercial serial MPP 3D printer. In that work, they used Gaussian-process (GP) regression to predict the height, radius, and volume of hemispheres and lines as a function of their location within the print volume. They then used the GP model to generate spatially dependent 3D models that compensate for the variation in height and radius inherent to the print volume. That work, however, focuses on inter-structure variation and compensation rather than intra-structure defects. Though this compensation can be valuable, for structures where the printing process leads to variations within the structure, such as a loss of features due to shrinkage^25^ or oxygen inhibition^26^, further work is needed to correct these errors. These intra-structure defects are particularly significant in micro-scale photopolymerization and are corrected by the methodology presented here in this work.

While neural network methods are often preferred for high dimensional datasets (large number of inputs or outputs), data-driven methods, like GP regression, will typically perform better for lower dimensional datasets when less training data is available. Furthermore, Bayesian ML models, like GP regression, provide a predicted value and a predicted variance^27^. The additional probabilistic information on the predicted variance means the ML model can guide the search for optimal parameters. This is a well-studied method known as Bayesian optimization (BO)^28^. BO has been proven to be effective for macro-scale 3D printing both for optimization of geometric accuracy^14^ and for optimization of part features for specific applications^29,30^. It has also been utilized to optimize the mechanical properties of micro-scale parts within finite element analysis simulations, which were later printed using MPP^31^. In this work, we demonstrate the effectiveness of BO for experimental design of 3D printing at the micro-scale. A BO framework is developed for micro-scale 3D printing to efficiently train a GP regression model to predict the geometric accuracy of patterns printed using PMPL. Two improvements are added to the Bayesian optimization scheme to further reduce data requirements: a multi-output GP regression model and an early stopping loop. Since PMPL is a layer-by-layer process, where an image is projected for each layer, 3D geometric accuracy is inherently determined by the 2D accuracy of each layer. Thus, the model is used for determining optimal binary image patterns for accurate printing of 2D shapes. We develop a novel parameterization scheme of input and output parameters that allow for the capture and correction of the prevailing defects in projection micro 3D printing. An optical data collection scheme is used where data is printed and measured using the PMPL experimental setup, thereby avoiding slow and costly measurements, such as the scanning electron microscopes used in most MPP studies. A multi-output GP model is trained using this experimental data from the PMPL process. Then, utilizing the probabilistic properties of the GP model to inform an acquisition function, we further improve the accuracy of the model by collecting additional data points from parts of the experimental parameter space that are most likely to lead to an improved model. The framework is demonstrated for three 2D shapes at three different scales: circles, squares, and triangles with outer dimensions of 27 µm, 13.5 µm, and 6.75 µm. We show that this BO framework is able to achieve greater accuracy than a typical CNN structure trained on a significantly larger dataset. The remainder of this paper describes the BO framework, detailing each step in the general order in which they take place within the process, and the experimental results of the process implementation. Finally, we discuss the generalization of the framework to other shapes through the concept of shape primitives^32^, its extension to the third dimension, and the broader implications of our framework and the parameterization scheme for other micro and macro-scale 3D printing processes.

## Results

### Bayesian optimization framework

**Fig. 1:**
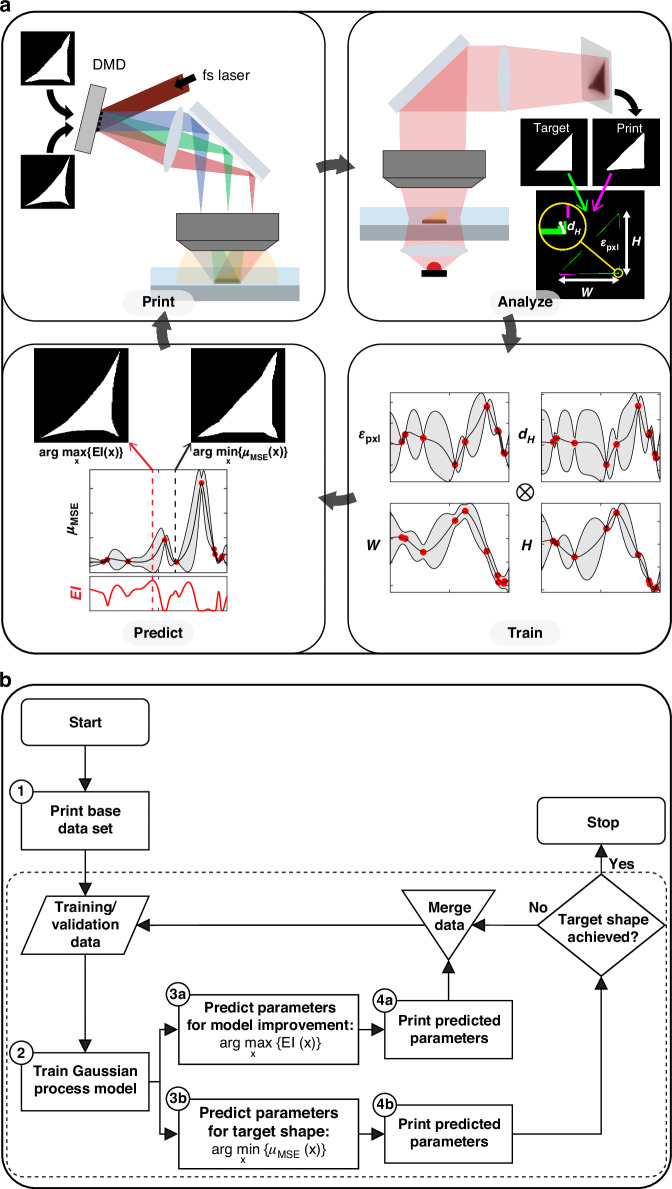
**Schematic and flowchart for the Bayesian optimization framework**. **a** Schematic of the Bayesian optimization framework applied to projection multi-photon lithography. **b** Flowchart for the Bayesian optimization framework. The process starts with the printing and analysis of a base data set uniformly distributed across the input parameter space. Step 2, the multi-output GP model is trained on the data set. In Step 3a the parameters for model improvement are predicted by maximizing the Expected Improvement, ${\rm{EI}}({\bf{x}})$. In Step 3b, the parameters for the early stopping criterion (achieving the target shape) are predicted by minimizing the shape error, ${\mu }_{{\rm{MSE}}}$. Then, in Step 4, the results for the predicted parameters are experimentally obtained and analyzed. If the stopping criterion is not met, the newly collected data is added to the training data and Steps 2-4 are repeated. The dashed frame encompasses the steps of the framework that are performed iteratively

A schematic and flowchart for the optimization process presented in this work are shown in Fig. 1. The major steps in the process are numbered in the flowchart. Here, we will provide a brief description of the general method as it can be applied to any printing process. In subsequent sections, we will provide a detailed explanation of the framework in the context of the specific shapes printed using PMPL. The process begins by collecting a base dataset for training the GP regression model (Step 1). This dataset is experimentally obtained for a uniformly distributed set of quasi-random points in the parameter space, which are selected using a Sobol sequence^33^, a type of low-discrepancy sequence used for sampling spaces more uniformly than truly random sampling. The results are then parameterized into the minimum number of output parameters capable of capturing the features that are to be optimized. After dividing the training data into the conventional 80:20 random split for training and validation, the GP model is trained to function as a surrogate for the experiment: providing a predicted set of output parameters for a given set of input parameters (Step 2). Now BO can be performed using the surrogate. In BO, an acquisition function takes in a mean and variance predicted by the surrogate at a given point in the parameter space and provides a value that indicates how desirable obtaining additional training data at that point is expected to be for the optimization process. This acquisition function can then be optimized using standard numerical methods to determine a set of input parameters that are most desirable for improving the GP model (Step 3a). The acquisition function used in this work (to be described later) is the Expected Improvement (EI) function^28^. Once a point (or points) that optimizes the acquisition function is identified, it is experimentally obtained and added to the training data (Step 4). This process is repeated to improve the accuracy of the GP model with each iteration. In conjunction with the BO loop, a stopping criterion loop is performed to determine when the GP model can identify a parameter set that provides sufficiently accurate results (Step 3b). For each iteration, experimental results from both loops are merged and added to the training/validation data. Typically, the acquisition function in BO will converge to the optimal parameters, however, using the stopping loop can allow for earlier termination, while BO would continue to explore other areas of the parameter space.

### Model parameters and data collection

In PMPL, high-intensity laser light is patterned by a DMD to form an image of the structure layer in a photosensitive resin. Each of the over one million micro-mirrors of the DMD is individually computer-controlled to tilt into the ON (reflected into the photoresist) or OFF (not reflected into the photoresist) position to emulate the white and black pixels of a digital image. The PMPL setup used in this work is described in Material and Methods and in Supplementary Note 1. Varying the images used to control the DMD is the key tool for improving the geometric accuracy of PMPL. For optimization of these images, five input parameters were chosen: width, height, radial warp, corner warp distance, and corner warp curvature (the latter three will be explained below). Each of the five input parameters is used to alter a target shape in the image sent to the DMD for printing. A study on the effects of the laser power and DMD exposure time is presented in Supplementary Note 2. It was determined that for PMPL, these parameters exhibit a strong threshold behavior, where above a certain threshold dose (power × exposure time) the results have little variation. As a result, the laser power and exposure time are held fixed within this study. Once the DMD patterns are printed using PMPL and developed, they are returned to the PMPL setup and imaged using a brightfield illumination setup. The effects of the input parameters on the printed shape are measured using image analysis of the brightfield images. Four parameters were chosen to quantify the quality of the printed shape relative to their target shape: pixel error, Hausdorff distance (to be explained below), width, and height.

**Fig. 2:**
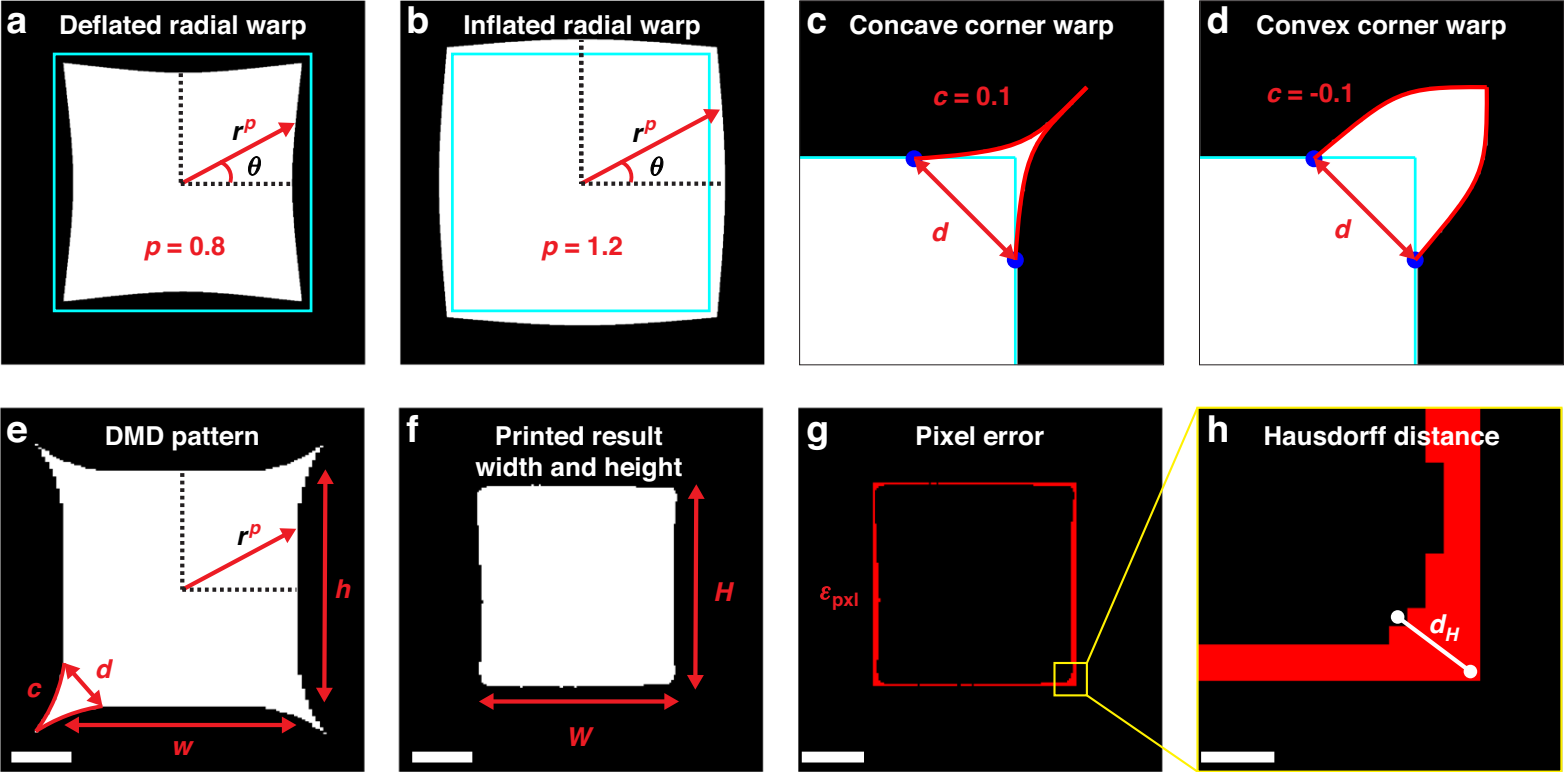
**Input parameters and output parameters**. The input parameters are shown in (**a**–**e**). The output parameters are shown in (**f**–**h**). The width, *w*, and height, *h*, input parameters which simply scale the pattern dimensions are not shown. The original unaltered shape boundary is denoted by a solid cyan line in (**a**–**d**). Radial warp, *p*, warps the shape according to a transformation from cartesian to radial coordinates with radius to the power *p*. **a** Radial warp of *p* < 1 “deflates” the shape. **b** Radial warp of *p* > 1 “inflates” the shape. Corner warping distance, *d*, defines the distance between the two points along the shape boundary. These points and the new corner vertex are all equidistant from the original corner and are dependent on *d*. The shape of the warped corner is controlled by the corner warping curvature, *c*. **c** Curvatures of *c* > 1 add a concave shape to the corner. **d** Curvatures of *c* < 1 add a convex shape to the *c*orner. **e** DMD pattern created using *w* = 7.853 µm, *h* = 7.849 µm, *p* = 0.997, *d* = 1.961 µm, and *c* = 0.308. **f** Width, *W*, and height, *H*, output parameters measured from the bounding box of printed result of pattern in (**e**). **g** Pixel error, *ε*_pxl_, of printed result is calculated by rescaling the target shape to match the area of the binarized printed shape, aligning the shapes’ centroids, counting the number of mismatched pixels, (shown in red in **g**) and dividing by the total number of pixels in the printed shape. **h** Hausdorff distance, *d*_*H*_, is the furthest minimum distance between any point on the transformed target boundary and the binarized printed shape boundary. The scale bars in **e**–**g** are 2 µm in length, and the scale bar in **h** is 250 nm in length

The input parameters are shown in Fig. 2, using a square as an example. The width, *w*, and height, *h*, parameters simply scale the size of the shape and are not shown in Fig. 2. Variations of these parameters are used to account for under-printing due to oxygen inhibition. The MPP process is known to be strongly dependent on oxygen concentration, where polymerization only occurs when oxygen is depleted^18,19^. For projection printing methods, oxygen inhibition can lead to under-polymerization at structure edges where oxygen can quickly diffuse from the surrounding non-depleted supply, whereas oxygen within the structure is consumed due to radical generation^6,10,26^. Figure 2a, b show the effect of the radial warp parameter, *p*, at *p* = 0.8 and *p* = 1.2, respectively. Radial warp is used to account for nonuniformity due to variation in polymerization and subsequent shrinkage. When applying the warp, the pattern undergoes a scaling transformation from the original shape’s cartesian coordinates to a radial coordinate system with $r\approx 1$ at the shape boundary. Then this radial coordinate system is transformed into a new nonlinear radial coordinate system where the radius is raised to an exponential power *p*, or$[x,y]\Rightarrow [{r}^{p}\,\cos \theta ,{r}^{p}\,\sin \theta ]$. For warp values larger than one the structure is “inflated”, and for warp values less than one the structure is “deflated”.

Another challenge common to projection printing is the loss of sharp features due to diffraction and oxygen inhibition effects. For multi-photon projection printing, these effects are even more apparent since the printed structures have features significantly closer to the length scales of the effects. In macro-scale SLA, several methods have been presented for improving the sharpness of features, both through ML^12^ and simpler data-driven methods^34,35^. For nanoscale photolithography, significant effort has been made to improve sharp features through optical proximity correction^36,37^. These resolution enhancement techniques for macro-scale SLA will often attempt to account for the larger pixel size at the print plane, while in photolithography, optical proximity correction typically focuses on reducing the effects of optical diffraction. For micro-scale projection printing, the dominant effect is chemical oxygen inhibition. Yet, each arrives at a similar conclusion, that an additional dosage of light is necessary around sharp, convex features. Here we achieve this using two parameters, corner warping distance, *d*, and corner warping curvature, *c*, as shown in Fig. 2c, d. A more detailed description of the corner warping method is provided in Supplementary Note 3.

Figure 2e shows an example of a DMD pattern created using values of *w* = 7.853 µm, *h* = 7.849 µm, *p* = 0.997, *d* = 1.961 µm, and *c* = 0.308 for the five input parameters. Figure 2f, g show the measurement of the output parameters on the binarized image of the printed result of this pattern in Fig. 2e. The output parameters are measured by image analysis of brightfield microscopy images of the printed results and binarized using custom image analysis programs written in Python primarily using the OpenCV package^38^. The threshold values used for binarization are determined using Otsu’s method^39^, which selects the threshold that maximizes inter-class variance between the foreground and background pixel distributions in the bimodal histogram of the grayscale image. A study on the accuracy of this optical data measurement system is presented in Supplementary Note 4. It was found that the measurement system is accurate to within 200 nm, just below the ~260 nm 2D feature size of the PMPL system. The width, *W*, and height, *H*, of the printed result are taken from the bounding box of the binarized shape, as shown in Fig. 2f. A pixel error measurement is taken to provide a scale-invariant measurement of the quality of the entire shape relative to the target shape. Figure 2g shows the shape from Fig. 2f compared with the square target shape. The target shape is scaled to match the area of the printed shape, the centroids are overlapped, and the number of pixels that do not match are counted. Figure 2g shows these mismatched pixels in red. The pixel error, *ε*_pxl_, is the number of mismatched pixels divided by the area of the printed shape. The last output parameter is the Hausdorff distance^40^, shown in Fig. 2h. The Hausdorff distance, *d*_*H*_, is the furthest minimum distance between any point on the printed shape’s boundary and any point on the transformed target shape’s boundary. Since this furthest distance typically occurs at the corner locations, like that seen in Fig. 2h, Hausdorff distance can be used to evaluate the quality of corner sharpness.

### Gaussian process regression model

The surrogate model used in this work is a GP-regression ML model^27^. A Gaussian process is a generalization of a Gaussian probability distribution, where it provides a distribution of functions rather than vectors. Just as a Gaussian probability distribution is specified by a mean and covariance value, a Gaussian process is specified by a mean function, $m({\bf{x}})$, and a covariance function, $k({\bf{x}},{\bf{x}}^{{{\prime} }})$. Using the GP formulation, we can predict a probability distribution for a set of functions that fit our training data. From this distribution, we can predict the mean, $\mu$, and standard deviation, $\sigma$, for each of the output parameters, ${\bf{y}}$, at a specific location, ${\bf{x}}$, in the input parameter space. The general form of these solutions is shown in Supplementary Note 5. The mean and covariance functions are our tools for shaping the scale and smoothness of the function distribution output by the GP. In this work, we selected a linear mean function, $m({\bf{x}})=A{\bf{x}}+b$, with weights *A* and bias *b* for hyperparameters, and a Matern covariance function^27^, with scale factor ${\sigma }_{k}$ and length scale ${l}_{k}$ hyperparameters. The hyperparameters are optimized using standard gradient descent learning methods (Adam optimization) to maximize the log marginal likelihood loss function. Often in GP regression, one will train a separate model for each output, also called a task. However, for a set of output parameters that are correlated, like ours, we can improve performance by allowing each output parameter’s covariance function to learn from the other outputs. To do this, we used a Kronecker-style multi-output GP regression model^41^. More details on the Matern kernel, the loss function, and the Kronecker-style multi-task formulation are given in Supplementary Note 5. The practical implementation of our GP model was done in Python using the GPyTorch^42^ package, a common open-source package for GP regression that is built upon PyTorch^43^.

### Bayesian optimization

After collecting a base dataset and training the GP regression model to predict the mean, $\mu$, and the standard deviation, $\sigma$, of the four output parameters, $[{\varepsilon }_{{\rm{pxl}}},{d}_{H},W,H]$, for a given set of the five input parameters, $[w,h,p,d,c]$, BO is performed to improve the GP model and achieve the target shape by maximizing the EI acquisition function (described in detail in Supplementary Note 5). In general, the EI is maximized at a point where the GP model expects a small mean error but has a very large standard deviation due to the lack of data in that location. The GP model outputs four means and standard deviations, but the BO acquisition function expects scalar mean and standard deviation values. To rectify this, we define a shape error, ${\mu }_{{\rm{MSE}}}$, using a weighted mean squared error function and from it a standard deviation, ${\sigma }_{{\rm{MSE}}}$, using propagation of uncertainty formulas:

1 $${\mu }_{{\rm{MSE}}}({\bf{x}})=\frac{1}{n}\mathop{\sum }\limits_{i=1}^{n}{w}_{i}{({\bar{\mu }}_{i}({\bf{x}})-{\bar{T}}_{i})}^{2}$$

2 $$\begin{array}{lll}{\sigma }_{{\rm{MSE}}}({\bf{x}})&=& \sqrt{\mathop{\sum }\limits_{i=1}^{n}{\left[{\bar{\sigma }}_{i}\frac{\partial }{\partial {\bar{\mu }}_{i}}({\mu }_{{\rm{MSE}}})\right]}^{2}}\\&=& \sqrt{\frac{1}{n}\mathop{\sum }\limits_{i=1}^{n}{\left[2{w}_{i}{\bar{\sigma }}_{i}({\bar{\mu }}_{i}({\bf{x}})-{\bar{T}}_{i})\right]}^{2}}\end{array}$$

Here, $n=4$ is the number of outputs of the GP model, ${\bar{\mu }}_{i}({\bf{x}})$ and ${\bar{\sigma }}_{i}({\bf{x}})$ are the mean and standard deviation value of the *i*th output of the GP model, ${w}_{i}$ is the weight value for the *i*th output, and ${\bar{T}}_{i}$ is the target value for *i*th output. The input and output data are z-score normalized for training the GP model, meaning they are scaled and shifted to have a standard deviation of one and a mean of zero. The vinculum over each symbol denotes that the value is the normalized value for that parameter. The unnormalized target values for pixel error and Hausdorff distance are always zero, while the width and height targets can be set to any desired size. The weight vector can be used to prioritize the optimization of one output parameter over another.

Using ${\mu }_{{\rm{MSE}}}$ and ${\sigma }_{{\rm{MSE}}}$, we can maximize the EI, ${\rm{EI}}({\bf{x}})$, with a standard numerical method. Maximizing the EI tells us what input parameters should be used during our next experiment (i.e., $\text{arg}\,\mathop{\max }\limits_{{\bf{x}}}\{{\rm{EI}}({\bf{x}})\}$) to improve the accuracy of the model. Simultaneous to BO, we can check the accuracy of the model by printing samples at the input parameter locations that the model expects to provide the minimum shape error, regardless of their EI for the model. This is our early stopping criterion. These parameters can be determined simply by minimizing the shape error given by Eq. 1, or $\text{arg}\,\mathop{\min }\limits_{{\bf{x}}}\{{\mu }_{{\rm{MSE}}}({\bf{x}})\}$.

### Optimization results

The Bayesian optimization framework was used in this work to optimize 2D circle, square, and isosceles right triangle patterns with target dimensions of 27 µm, 13.5 µm, and 6.75 µm. Due to the high printing rate of PMPL, many samples can be quickly printed on one substrate for each trial/iteration of the BO process. A base dataset of 512 samples was collected for the 6.75-µm shapes, and a base dataset of 256 samples was collected for the 13.5-µm and 27-µm shapes. As will be seen below, the smaller shapes have larger deviations from the target shape and benefit from more training data for greater accuracy. An alternative method which may reduce data requirements further, is to use smaller base data sets and instead perform more BO. However, due to the high-speed nature of PMPL and ex-situ data collection, it is more efficient to begin with a relatively larger base dataset to reduce experimentation. For each of the subsequent trials of BO, multi-batch acquisition was performed to ensure global optimization. The maximum of the EI, $\text{arg}\,\mathop{\max }\limits_{{\bf{x}}}\{{\rm{EI}}({\bf{x}})\}$ was found from 128 uniformly distributed starting points in the input parameter space. Then, any redundant sets of optimal parameters were removed, and the remaining sets were printed and measured one time each. For the early stopping criterion, $\text{arg}\,\mathop{\min }\limits_{{\bf{x}}}\{{\mu }_{{\rm{MSE}}}({\bf{x}})\}$ was found from 64 uniformly distributed starting points. Then, the minimum eight points were printed ten times each and measured. Throughout the trials of BO, the entire dataset was randomly split 80/20 into training and validation sets. After completion of the optimization, the 6.75-µm shapes had 621, 736, and 820 data points for the circle, square, and triangle shapes, respectively. The 13.5-µm shapes had 339, 482, and 454 data points, and the 27-µm shapes had 396, 503, and 470 data points for the circle, square, and triangle shapes, respectively.

**Fig. 3:**
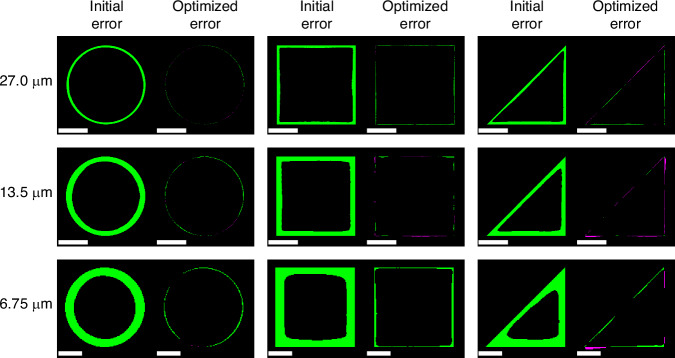
**Comparison of initial and optimized printed results**. The mismatched pixels between the target shape centered and overlapped with the binarized printed results of the initial unaltered target pattern (left) and optimized pattern (right). Green pixels show where the printed structure is smaller than the target shape. Magenta pixels show where the printed structure is larger than the target shape. The top, middle, and bottom rows show the 27-µm, 13.5-µm, and 6.75-µm results, respectively. The scale bars are 10 µm, 5 µm, and 2 µm for the top, middle, and bottom rows, respectively

The qualitative results of the nine different tests are shown in Fig. 3. Each image shows the mismatched pixels from a binarized printed structure centered and overlapped with the target pattern. No rescaling was done for any shape in order to evaluate the accuracy of both the contour and scale of the printed shape relative to the target. Locations where the printed shape is undersized (within the target pattern) are shown in green and areas where the printed shape is oversized (outside the target pattern) are shown in magenta. The initial printed results when the target pattern was printed directly are shown on the left of each subfigure. The printed results of an optimized pattern determined by the framework are shown on the right of each subfigure. The top row shows the 27-µm results, the middle row shows the 13.5-µm results, and the bottom row shows the 6.75-µm results.

For each size, printing the unaltered target pattern led to a printed structure that was consistently undersized by ~1.4 µm due to an oxygen dead zone around the edge of the structure. The BO framework, in all nine cases, compensated for this effect and improved corner sharpness, reducing the shape error within just 4 iterations. Table 1 shows the perimeter error (area of mismatched pixels in Fig. 3 divided by the perimeter of the target shape) in units of µm for the initial and optimized results for all the tests. The perimeter error represents an average error along the perimeter of the printed structure. For each case, the perimeter error is reduced from between ~0.5–1 µm to 116 nm or less. The resulting errors are all within the determined measurement accuracy and are roughly equivalent to 1–2 of the 62 nm demagnified camera pixels.

**Table 1:** Results of perimeter error reduction by Bayesian optimization

Target Size	Circle		Square		Triangle	
	Initial (µm)	Optimized (µm)	Initial (µm)	Optimized (µm)	Initial (µm)	Optimized (µm)
27.0 µm	0.712	0.038	0.928	0.099	0.765	0.085
13.5 µm	0.858	0.049	0.787	0.038	0.623	0.056
6.75 µm	0.647	0.051	0.627	0.116	0.494	0.054

**Fig. 4:**
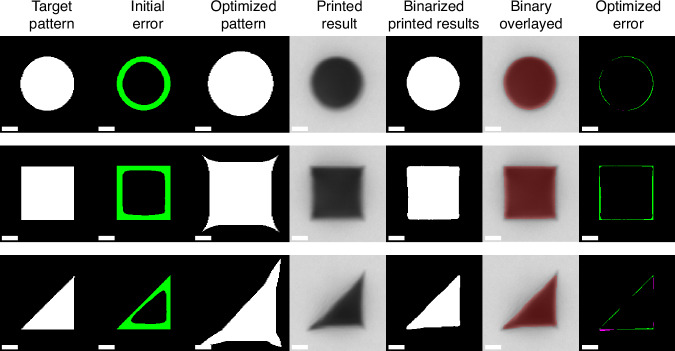
**Results for 6.75-µm shapes**. Each row contains the results for each of the 6.75-µm shapes. Column 1 shows the target pattern. Column 2 shows the printed result of the target pattern compared with the target shape. Column 3 shows the optimal pattern determined by the framework. Column 4 shows the printed result of the optimal pattern. Column 5 shows the binary shape detected in the grayscale image. Column 6 shows the binary shape overlayed on the grayscale image. Column 7 shows the printed results of the optimal pattern compared with the target shape. The scale bars in all images are 2 µm

The smallest, 6.75-µm, shapes proved to be the most challenging for the framework. Despite this, the 6.75-µm results show a significant improvement after optimization. The detailed image-based results for optimization of the three 6.75-µm shapes are shown in Fig. 4. The qualitative and quantitative results for all the nine test cases are shown in Supplementary Note 6, organized by shape rather than size. In Fig. 4, each row contains the results for each of the 6.75-µm shapes. All images are to scale, with a 2 µm long scale bar in each. The first column shows the target pattern. Column two shows the printed result when printing this target pattern (no BO) and compares it with the target. The third column shows the optimal pattern determined by the framework after four iterations of BO. The printed results of these patterns are shown in column four. Column five shows the detected binary shape from the grayscale image. To observe the relationship between the grayscale and binary images, the second to last column shows the binary shape overlayed in red with the grayscale image. Finally, the last column shows the comparison of the optimal printed result with the target shape.

The optimal DMD patterns shown in Fig. 4 display some similarities. Each pattern was scaled up from 6.75 µm to dimensions around 7.7–7.9 µm. The square and triangle had optimal corner warping distances of *d* = 1.91 µm and *d* = 1.53 µm, respectively. Although a significant amount of additional area was added to the perimeter and corners of the patterns, only a portion of the interior overcame oxygen inhibition and polymerized, leading to the improved accuracy and corner sharpness seen in the results of Fig. 4. The optimized error results in Fig. 4 do show some artifacts of the unconventional optimized patterns, with the appearance of several rounded edges near sharp features. Interestingly, these artifacts are similar to those observed in SLA^12,34^ and nanolithography^36^ resolution enhancement techniques, suggesting these artifacts may be inherent to the minimization of error/enhancement of sharp features in photopolymerization. It is possible these artifacts could be further reduced by additional corner warping parameters for more degrees of freedom. However, these artifacts still lie within the accepted 60–120 nm (1–2 camera pixel) error range for this study. The BO framework was also tested on other geometries, as shown in Supplementary Note 7. The model proved to be equally effective for a 6.75 µm pentagon and 5-pointed star. The framework was able to achieve perimeter errors of 0.049 µm and 0.064 µm, for the pentagon and star, respectively.

Since few ML optimization schemes have been presented for MPP, especially for PMPL, it is difficult to compare the BO framework presented in this work to other existing literature. For comparison, we have trained a typical convolutional neural network structure on the nine base datasets of the BO framework. The details and results of this CNN are presented in Supplementary Note 8. The CNN is an autoencoder structure that takes an image input and provides a predicted image output. We have trained the CNN as an inverse model to provide a predicted DMD pattern necessary to achieve the desired input printed result. The CNN was used to predict the optimal DMD patterns for the 6.75-µm target shapes, and the patterns were then printed and analyzed using the same optical measurement scheme as the BO framework. Despite having 3067 training datapoints, about 4–5 times larger than the BO test cases’ datasets, the perimeter errors of the CNN predictions were two to five times larger than the 6.75-µm BO results.

## Discussion

A major benefit of GP-regression-based ML is its versatility due to the limited assumptions made about the functional form of the data. Additionally, the multi-input, multi-output nature of the multi-task GP model presented here provides greater flexibility in designing the parameterization scheme, owing to its inherent ability to accommodate multidimensional input and output parameter configurations. Consequently, the framework presented here can easily adapt to alternative or additional parameters or to entirely new parameter spaces of different manufacturing processes. For optimization of PMPL, the most obvious next steps are extension to other shapes and to 3D shapes. To generalize models with parametric representations of shapes, one promising avenue is the piecewise combination of shape primitives to represent complex shapes^32^. With this method, complex shapes are constructed from the superposition of simpler primitive shapes for which comprehensive models have already been built, such as those presented in this work. The extension of our framework to the third dimension is primarily limited by the data collection scheme. There has been exciting recent work to establish in-situ 3D measurement techniques for structures in MPP^44,45^. The combination of in-situ data collection with the framework presented here only amplifies its impact. The input and output parameters presented here can be extended to the 3D, and if necessary additional parameters can be easily added to the framework. While additional parameters could increase data requirements, this would likely be offset by the convenience and efficiency of in-situ data collection.

ML has been a focus in the AM field in recent years and has been studied in macro-scale applications. However, the unique challenges that MPP presents mean a direct analog from these works to MPP is not guaranteed. Challenges such as measuring micro and nanoscale features in a cost-effective manner to generate sufficient training data or combating chemical processes such as oxygen inhibition (which now operate on length scales significantly closer to the desired feature sizes) require new ML solutions. We presented a set of parameters that can be leveraged to analyze and combat the challenges common to many MPP processes and also those unique to the more recent projection-based processes. The challenge of under-printing due to shrinkage and oxygen inhibition, both uniform and nonuniform, is universal to MPP. The scaling and warping parameters in our framework proved effective in addressing these issues. The retention of sharp features is another challenge faced by any 3D printing process. The new corner warping scheme developed in this work successfully improved corner sharpness at multiple scales for multiple shapes. These methods could easily be translated to other 3D printing processes, including the serial, line-by-line MPP process by slicing the images into individual paths. Although corner sharpness in MPP has typically been directly dependent on the linewidth, the more recent introduction of grayscale serial MPP^21^ has obscured this relationship, potentially creating the opportunity for ML-based corner warping like that shown here.

With the continued advances in additive manufacturing, especially MPP, ML frameworks will be valuable. The ongoing push in MPP to expand the library of printable materials, improve resolution, increase throughput, and in general broaden its applications^46^ means that more and more new design spaces will need to be explored. Our framework for optimizing process parameters for a specific printing objective can provide an efficient way to guide the exploration of these spaces. The ML framework we introduce in this work is an active ML framework demonstrated on the optimization of print patterns in high-speed layer-by-layer projection multi-photon 3D printing. The framework employs Bayesian optimization to strategically collect the most informative data, enhancing the training of a GP regression-based model. The multi-output GP model leverages correlations between the process parameters, thereby improving accuracy with less data and providing uncertainty quantification to guide the optimization. This model acts as a surrogate for the manufacturing process, predicting optimal parameters for desired geometries. The addition of an early stopping criterion is used to reduce data collection efforts by terminating the Bayesian optimization earlier than typical methods. Testing on three 2D shapes at different scales demonstrates that the framework significantly improves geometric accuracy, reducing errors within measurement accuracy in just four optimization iterations. We showed that with as few as 339 training data points and only 4 iterations, or 5 experiments, our BO framework can be employed to improve the accuracy of the novel projection multi-photon lithography process. The success of these case studies and the adaptability of the multi-output GP regression-based surrogate model suggest the framework’s broad applicability to other AM processes, enhancing accuracy with less experimental effort.

## Materials and methods

### Materials

All chemicals were used as they were received. 4-(Dibutylamino) benzaldehyde, 4-methylcyclohexanone, potassium hydroxide, and pentaerythritol triacrylate (PETA) were purchased from Sigma-Aldrich. Isopropanol and acetone were purchased from JT Baker. SU-8 Developer was purchased from Kayaku Advanced Materials.

### Synthesis and preparation of photoresists

Synthesis of the photoinitiator (2E,6E)-2,6-Bis (4-(dibutylamino) benzylidene)-4-methylcyclohexanone (BBK) was done according to the procedure reported previously, via an aldol condensation reaction^10,47^. Photoresist mixtures were created by mixing the photoinitiator BBK with the monomer PETA at 0.38 mol% concentration and sonicating for 4 h.

### Preparation of printing substrate

Photoresist mixtures were drop-casted onto 1 mm thick water-white glass microscope slides (Fisher Scientific Cat. No. 125441). Prior to the drop-casting of the photoresist, the microscope slides were precleaned. The slides were first wiped with acetone and lint-free task wipes. Then, the slides were sonicated in a bath of Alconox and ultra-pure water mixture for 5 min. Next, the slides were rinsed with ultra-pure water and sonicated in an acetone bath for 5 min. Finally, they were rinsed with ultra-pure water and dried with nitrogen.

### 3D printing system

Supplementary Note 1 provides a detailed description of the PMPL experimental setup. The PMPL setup uses an ultrafast Ti-sapphire regenerative amplifier laser to illuminate a DMD. The DMD is imaged within the photoresist by a 4f configured achromatic doublet lens and objective lens dipped into the photoresist. Sample positioning was done using an air-bearing stage. Samples with a target dimension of 6.75 µm were printed using a 1.49 NA 100× objective lens with an exposure time of 10 ms and an average power incident on the DMD of 340 mW. Samples with a target dimension of 13.5 µm and greater were printed using a 1.45 NA 60× objective lens with an exposure time of 10 ms and an average power of 600 mW.

### Post-print processing

After printing, samples were placed in a bath of SU-8 Developer for 20 min with a magnetic stir bar stirring at 1150 rpm. The samples were then transferred to an isopropanol bath for ~2 min and blow-dried with nitrogen. After development samples were sputter coated with an Au/Pd mixture to enhance contrast for subsequent brightfield transmission microscopy.

### Sample imaging

Imaging of the samples was done using the same system used for 3D printing, with the setup operating as a brightfield transmission illumination microscope. An Olympus 100× (NA = 0.9) air objective was used for the imaging process. A 660-nm LED illumination source (Thorlabs M660L3-C3) was focused at the sample plane using a Nikon 100 × ELWD objective lens (NA = 0.8). The imaging process was automated using LabVIEW and Aerotech’s NVIEW program.

## Supplementary information

Supplemental Material

## Data Availability

The data supporting the results within this paper and other findings of the study are available from the corresponding authors upon reasonable request. The custom computer codes used within this study are available from the corresponding authors upon reasonable request.
