# Supplementary material for: Bayesian optimization with Gaussian-process-based active machine learning for improvement of geometric accuracy in projection multi-photon 3D printing
Source: Light Sci Appl. 2025 Jan 20;14:56. doi: 10.1038/s41377-024-01707-8 (PMC11743787; doi:10.1038/s41377-024-01707-8)
Supplement: Supplementary file 1 — Supplemental Material [file 41377_2024_1707_MOESM1_ESM.pdf]

Supplementary Information for Bayesian Optimization with Gaussian-Process-Based Active Machine Learning for Improvement of Geometric Accuracy in Projection Multi-photon 3D Printing

Jason E. Johnson[1,2], Ishat Raihan Jamil[1,2], Liang Pan[1,2], Guang Lin[1,3], Xianfan Xu[1,2]*

[1]School of Mechanical Engineering, Purdue University, 585 Purdue Mall, West Lafayette, IN 47907, USA

[2]Birck Nanotechnology Center, Purdue University, 1205 Mitch Daniels Blvd., West Lafayette, IN 47907, USA

[3]Department of Mathematics, Purdue University, Mathematical Sciences Bldg, 150 N University St, West Lafayette, IN 47907, USA

Jason E. Johnson (john2811@purdue.edu)

Ishat Raihan Jamil (ijamil@purdue.edu)

Liang Pan (liangpan@purdue.edu)

Guang Lin (guanglin@purdue.edu)

*Corresponding author: Xianfan Xu (xxu@purdue.edu, (765) 494-5639)

Supplementary Note 1: Projection Multi-photon Lithography Experimental Setup

Figure S1 provides a simplified schematic of the experimental setup used for PMPL. The printing laser was a Ti-sapphire regenerative amplifier (Coherent Legend Elite Duo USX) with a ~50 fs pulse duration, 5 kHz repetition rate, 800 nm center wavelength, and 30 nm bandwidth. The power was controlled using a half-waveplate (Thorlabs AHWP10M-580) and a polarizing beam splitter cube (Thorlabs PBS25-780). A concave lens (f = -125 mm, Edmund Optics #49-539) and convex lens (f = 150 mm, Thorlabs LA1417-B) were used to collimate and expand the beam to a ~12 mm beam diameter. A piezo-actuated tip-tilt mirror (PI S-330.8SL) was used to circularly translate the beam at a high rate during printing to reduce the effects of laser speckles. The beam was shaped from a Gaussian intensity profile to a flattop profile with a πShaper (AdlOptica πShaper 12_12_TiS_HP). Then, the beam was directed onto the DMD (DLP4500NIR) at 24° from the DMD surface normal. An achromatic doublet (f = 180 mm, Thorlabs AC508-180-AB) collected the light from the mode diffracted along the DMD's normal axis and focused it at the back focal plane of the microscope objective lens (Nikon 100×, NA = 1.49, Olympus 60×, NA = 1.45). A dichroic mirror, reflective from 760 nm to 840 nm (Eskma Optics, 045-800), was used to redirect the printing laser 90° while allowing a helium-neon laser to transmit through for focus detection. A 50/50 beam splitter (Thorlabs BSW27) was placed after the dichroic mirror and before the objective lens for in-situ imaging of the sample plane with an imaging lens (f = 100 mm, Thorlabs LA1509-B) and a CMOS camera (FLIR GS3-U3-32S4M). The objective lens was dipped directly into the photoresist on the pre-cleaned microscope slide. Sample positioning was done using a 3-axis air bearing stage (Aerotech ABL1000 series). The printing of samples was automated via serial communication with the DMD controller through MATLAB and LabVIEW in combination with motion control via Aerotech's NVIEW program.

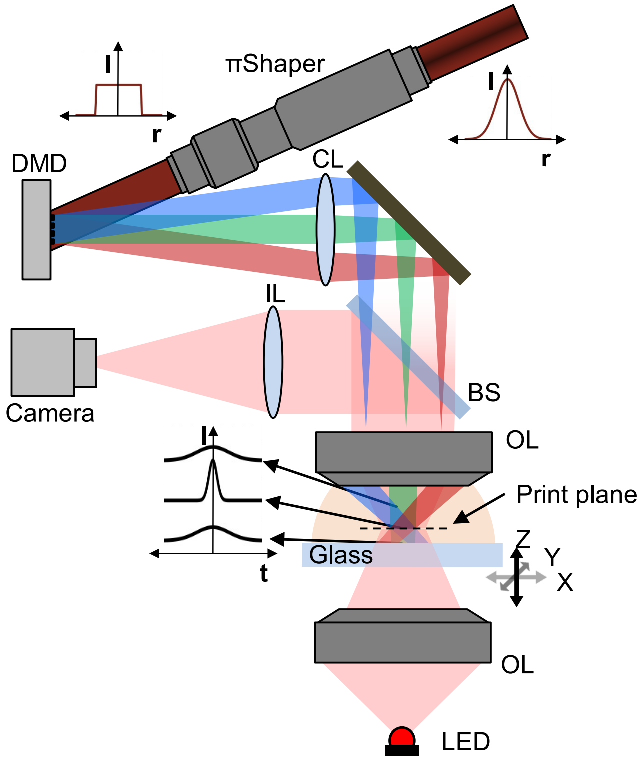

Fig. S1 Projection multi-photon lithography experimental setup. An 800 nm ultrafast amplified laser is passed through a πShaper to shape the Gaussian intensity profile into a flattop intensity profile. The beam illuminates a digital micromirror device (DMD). The grating-like nature of the DMD disperses the beam into its component wavelengths. A collecting lens (CL) and objective lens (OL) in a 4f configuration recombine the dispersed components at the print plane, which is submersed in a photoresist. A glass substrate is translated via a 3-axis stage during fabrication. A 660 nm LED is passed through a second objective lens (OL) to focus the light through the print plane for high NA illumination. A beam splitter (BS) is used to reflect the illumination light through an imaging lens (IL) onto a CMOS camera for monitoring of printing and, after development, imaging of printed structures.

Supplementary Note 2: Study of the Effects of Laser Power and Exposure Time

The laser power or intensity and DMD pattern exposure time are two parameters which are fixed for the results used in this work. While optimization of these parameters can be vital in many conditions, for 2D printing with PMPL, the results exhibit a strong threshold effect. Above a certain threshold dose (i.e. laser power × exposure time) the trends are relatively weak for the output parameters. To illustrate, a set of structures with input parameters uniformly distributed across the 6.75-µm input parameter space were printed at a range of laser powers and DMD exposure times. They were then analyzed and summarized using the shape error as defined in Eq. 1 of the main text. Figure S2a shows the μ_{MSE} results for each of the shapes and how they vary with dose. Above a certain dose, ~3 mW·s, the results have little change. Figure S2b presents the same results averaged at each power and exposure within a 2D heatmap. The lower left corner of the plot has large shape error, but once a threshold dose is reached the shape error has a relatively flat trend. As long as the power and exposure values remain above this threshold the framework can identify parameters which minimize shape error. The results of Supplementary Note 7 were obtained at a power of 540 mW and exposure time of 2 ms to confirm that the framework has similar effectiveness at powers and exposures other than the 340 mW and 10 ms values used in the main text. On the other hand, laser power and exposure time can be easily added to the model's input parameters for optimization of processes for which this is desirable. However, for our work, the weak trend towards higher doses for minimization of shape error, observable in Fig. S2, means the framework for PMPL will trend towards maximizing dose which is generally undesirable for rapid projection printing.

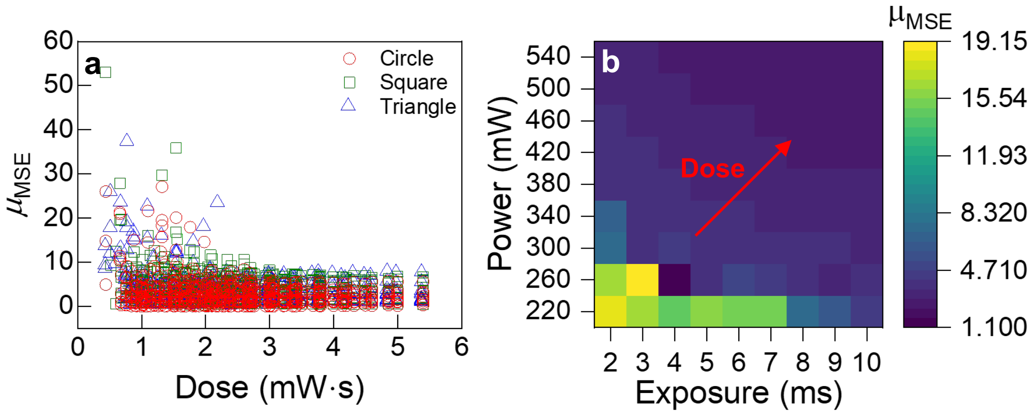

Fig. S2 Effects of laser power and DMD exposure time on printed results. a Variation of shape error, μ_{MSE}, with laser dose (power × exposure) for circle, square, and triangle patterns uniformly distributed about the 6.75-µm input parameter space. **b** Heat map of shape error as it varies with both laser power and DMD exposure time.

Supplementary Note 3: Corner Warping Method

The corner warping method used in this work was designed to provide the machine learning (ML) model with a general tool to improve corner sharpness. The method allows for a broad range of different types of geometries to be added to the corners of shapes. All pattern generation, including the corner warping, was done in MATLAB. Figure S3a shows the corner of an unaltered square pattern. The points used in the corner warping procedure and the corner boundaries for different values of corner warping curvature, *c*, are shown in Fig. S3a. First, the corner, (x_0, y_0), marked by an open red marker, is found using a customized MATLAB function[1]. Next, the solid red points, (x_1, y_1) and (x_2, y_2), are determined by selecting two points along the boundary of the shape equidistant from the corner and a distance *d* apart from each other. Then, the third extended point, (x_3, y_3), falls along the corner's bisecting line and is set at a distance such that all three

points are equidistant from the corner. Once the points are defined, the curve is determined by using a linear transformation to take a power law curve from a "function" space to the pixel space. The power law curve is of the form $y = a(x-b)^{-c} - 1$, where a and b are set such that the curve passes through (0,1) and (1,0). This relationship was chosen to allow for the creation of geometries like those used previously for SLA corner sharpness studies[2,3]. Figure S3b presents the power law curves for different curvature values in the "function" space. Figure S3c illustrates the curves after they have been transformed into the pixel space. The transformation matrix is determined by finding the linear transformation that translates the point at (0,1) to (x_1, y_1), and the point at (1,0) to (x_3, y_3), while keeping the corner, (x_0, y_0), fixed as the origin. Then the remainder of the points making up the curve are translated according to this transformation matrix. The black dotted line indicates the axis of reflection that is used to invert the curve when c is negative. The fully enclosed geometry is generated by repeating the process with (x_3, y_3) and the lower point on the boundary, (x_2, y_2).

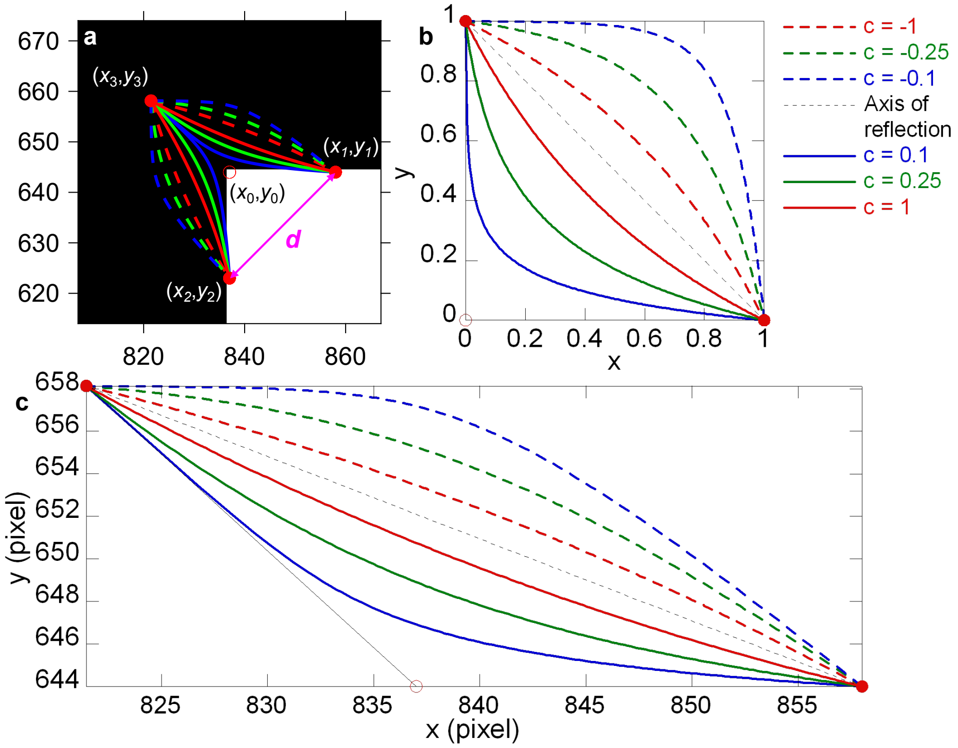

Fig. S3 Corner warping by linear transformation. a View of boundaries formed by different corner warping curvature, *c*, values on the corner of an unaltered square. All have a set corner warping distance *d*. **b** Power law curve, $y = a(x-b)^{-c} - 1$, in function space. Variables *a* and *b* are set such that the curve passes through (0,1) and (1,0). **c** Power law curves after undergoing linear transformation to map (0,1) and (1,0) in **b** to (x_1, y_1) and (x_3, y_3) in **a** and **c**. The open red marker denotes the corner that is used as the origin in the function space, **b**, and the pixel space, **c**.

Figure S4 provides an example of a triangle pattern with warped corners. This pattern has one set of corner warping parameters for all corners. However, the warping method was designed such that the two smaller angle corners have a much greater area added to the corner when

compared to the right angle. These sharper corners protrude further from the shape and therefore allow more additional area to combat the supply of oxygen along the shape boundary.

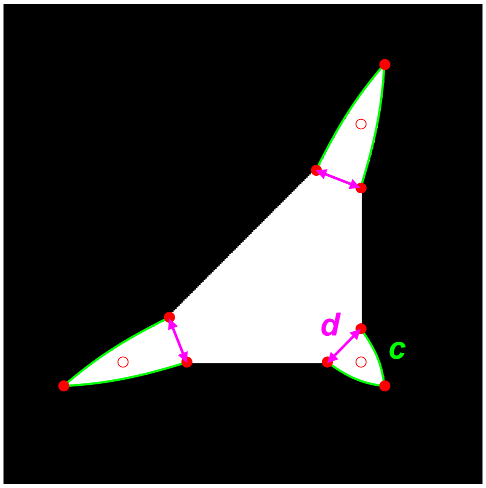

Fig. S4 Angle-independent corner warping parameters. For the same values of corner warping distance, *d*, and corner warping curvature, *c*, the area added scales inversely with the angle of the corner. The same *d* value is highlighted in magenta. The same *c* value is highlighted in green. The solid red points are the locations found during the corner warping process. The open red point is the original corner location.

Supplementary Note 4: Accuracy of PMPL Printing Process and Data Collection Measurements

The resolution and minimum feature size for the PMPL process have previously been reported in detail for 3D printing[4]. Additional studies were performed to demonstrate the feature size for 2D printing with PMPL. Figure S5 shows the results of these studies. To examine the minimum feature size, line arrays were printed, as shown in Figs. S5a and S5b. A minimum linewidth of 260 nm was obtained at a laser power of 360 mW and an exposure time of 25 ms. Also, an array of varying size holes was printed, as shown in Figs. S5c and S5d. A minimum hole size of ~550 nm was obtained. Since oxygen inhibition reduces polymerization, its presence

reduces the linewidth. Whereas for the converse, oxygen inhibition increases the minimum hole size. Hence the minimum feature size of the holes is larger than that of lines. The results of Fig. S5 were not optimized with the framework presented in the main text. Future work will inspect these hole sizes using higher resolution imaging methods and investigate optimization involving the use of a pre-exposure pattern to reduce oxygen within the holes prior to printing.

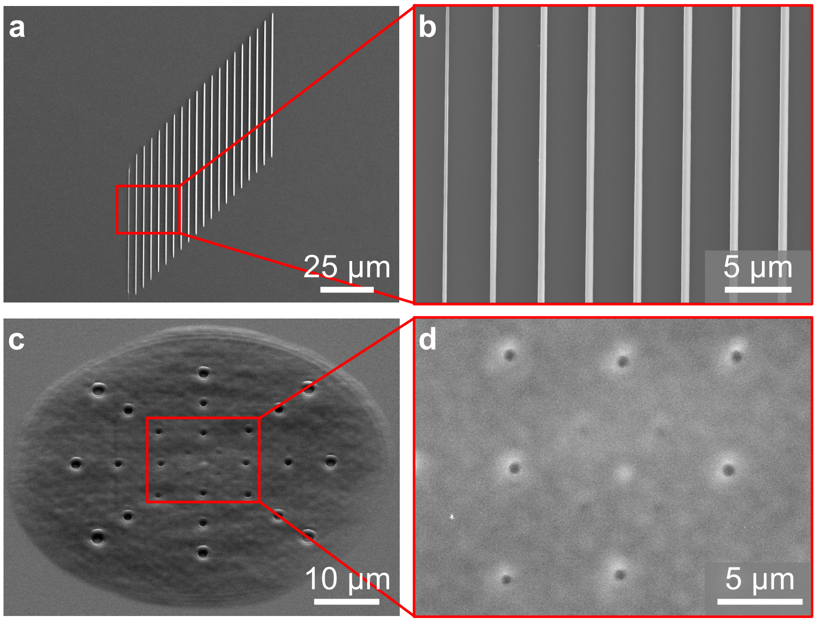

Fig. S5 Minimum feature size and hole size of 2D printing with PMPL. a Array of lines printed with varying exposure times. **b** Magnified view of printed lines with the leftmost line obtaining a linewidth/feature size of 260 nm. **c** Array of holes of varying size printed by PMPL. **d** Magnified view of innermost holes with diameters of ~550-700 nm.

The accuracy of the brightfield-microscopy-based measurement scheme was tested by comparing results with measurements from a scanning electron microscope (SEM). Circular structures with diameters from approximately 2-13 µm were printed using the PMPL setup. The structures were measured both via SEM and via the data analysis method presented and used in

the main text. Figure S6 shows the measurement error for the optical microscope images when compared with measurements for the SEM images. The measurement error was computed by averaging the bounding box width and height of the structures in the optical images and subtracting the average bounding box width and height from the SEM images. The typical error is approximately –200 nm, meaning the optical images slightly underestimate the structure size. As smaller structures are measured the diffraction limit begins to affect the optical measurement scheme, which overestimates the structure size. Since the 62 nm demagnified camera pixels are already oversampled, the 660 nm illumination wavelength can be reduced if greater accuracy were necessary.

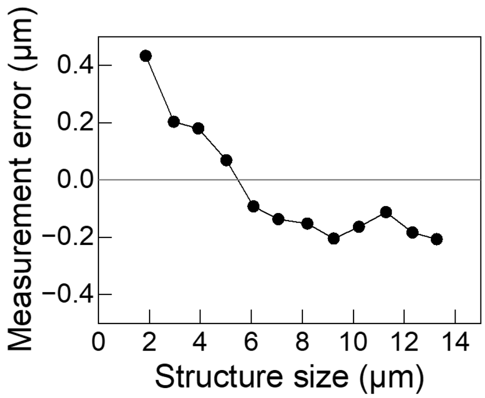

Fig. S6 Brightfield-microscopy-based measurement error. The difference between the size of structures determined via the brightfield-microscopy-based measurement scheme used in the main text and via SEM images for circular structures ranging from ~2-13 µm in diameter.

Three triangle structures with target outer dimensions of 2 µm, 3 µm, and 4 µm were optimized to test the minimum size structures that can be optimized using the optical data collection method. Table S1 shows the perimeter error results for these structures. For each size,

the observed perimeter error was reduced from an initial value of 225-340 nm down to 49-67 nm, similar to the larger triangles shown in Table 1 (54 – 85 nm).

Table S1. Perimeter error results for small triangle target shapes.

Target Size (μm)	Initial (μm)	BO (μm)
2	0.225	0.065
3	0.320	0.049
4	0.340	0.067

Figure S7 shows the qualitative results for the initial and optimized patterns. It shows the drastic improvement between the initial and optimized printing results.

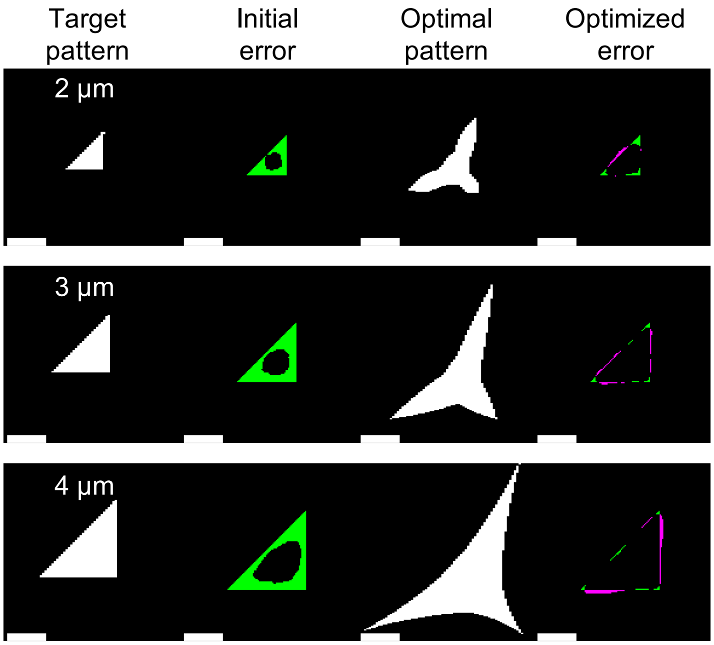

Fig. S7 Images for optimizing smaller triangle target shapes. Shown from left to right are the initial unaltered target pattern, the printed results of the initial pattern compared with the target shape, the optimized pattern determined by the BO framework, and the printed results of the optimized pattern compared with the target shape. Green pixels show where the printed structure is smaller than the target shape. Magenta pixels show where the printed structure is larger than the

target shape. The sizes are ordered by row from top to bottom are the 2-μm, 3-μm, and 4-μm results. The scale bar is 2 μm for all images.

Supplementary Note 5: Gaussian Process Regression and Bayesian Optimization

The goal of Gaussian process (GP) regression is to predict a continuous value y from an input vector $\mathbf{x}$ by mapping a finite set of training data to a function that predicts all possible inputs. Here we provide a description of how this is accomplished in GP regression. A more detailed treatment can be found in Rasmussen and Williams[5]. GPyTorch[6] was the Python package used for the inference described below.

Just as a Gaussian or normal distribution of vectors is specified by a mean and a covariance, $\mathcal{N}(\mu,\Sigma)$, a Gaussian process is a distribution of functions specified by a mean function and a covariance function, $\mathcal{GP}(m(\mathbf{x}),k(\mathbf{x},\mathbf{x}'))$. For GP regression, we will assume our observations follow the following relationship, $y = f(\mathbf{x}) + \varepsilon$, where ε is noise in our observations that follows a zero mean Gaussian distribution with variance σ_n^2, or $\varepsilon \sim \mathcal{N}(0,\sigma_n^2)$. Here, ~ is shorthand for saying the probability distribution of ε is equal to $\mathcal{N}$. The underlying function describing our process is $f(\mathbf{x})$, and its probability distribution is modeled by the GP, or $f(\mathbf{x}) \sim \mathcal{GP}(m(\mathbf{x}),k(\mathbf{x},\mathbf{x}'))$. The value y is called the likelihood. Using our training data set of observations for a set of $\mathbf{x}$, or $\{\mathbf{y}, X\}$, we can perform predictions by conditioning the joint Gaussian prior distribution on the observations according to Bayes theorem[5]. We can write the joint distribution of the training data, and the vector of function values, $\mathbf{f}_*$, at the test locations, X_*, (i.e. locations not in the training data) as,

$$\begin{bmatrix} \mathbf{y} \\ \mathbf{f}_* \end{bmatrix} \sim \mathcal{N}\left(\begin{bmatrix} \mathbf{m}(X) \\ \mathbf{m}(X_*) \end{bmatrix}, \begin{bmatrix} K(X,X)+\sigma_n^2 I & K(X,X_*) \\ K(X_*,X) & K(X_*,X_*) \end{bmatrix} \right), \quad \text{S1}$$

where $\mathbf{m}(\cdot)$ is the vector of values given by the mean function, or $\mathbf{m}_i = m(\mathbf{x}_i)$, $K(\cdot,\cdot)$ is the covariance matrix given by the values determined from the covariance function, or $K_{ij} = k(\mathbf{x}_i, \mathbf{x}_j)$, and I is the identity matrix. According to Bayes theorem, the probability for the posterior distribution, $\mathbf{f}_*$, for a test locations, X_*, is:

$$p(\mathbf{f}_* | X, \mathbf{y}, X_*) = \frac{p(\mathbf{y} | X, \mathbf{f}_*, X_*)\, p(\mathbf{f}_* | X_*)}{p(\mathbf{y} | X)}. \quad \text{S2}$$

From Eq. S2, and using some properties of multivariate Gaussian distributions, we can derive the predictive equations for Gaussian process regression, $\mathbf{f}_* | X, \mathbf{y}, X_* \sim \mathcal{N}(\bar{\mathbf{f}}_*, \mathrm{cov}(\mathbf{f}_*))$, where $\bar{\mathbf{f}}_*$ is the predicted mean function and $\mathrm{cov}(\mathbf{f}_*)$ is the predicted covariance function and they have the following form[5],

$$\bar{\mathbf{f}}_* = \mathbf{m}(X_*) + K(X_*, X)\left[K(X,X) + \sigma_n^2 I\right]^{-1}(\mathbf{y} - \mathbf{m}(X)), \quad \text{S3}$$

$$\mathrm{cov}(\mathbf{f}_*) = K(X_*, X_*) - K(X_*, X)\left[K(X,X) + \sigma_n^2 I\right]^{-1} K(X, X_*). \quad \text{S4}$$

These results provide the predicted mean and covariance for the noise free scenario, i.e. $f(\mathbf{x})$ in $y = f(\mathbf{x}) + \varepsilon$. We are interested in predicting the likelihood y. Therefore the mean values, μ, discussed in the main text are taken directly from $\bar{\mathbf{f}}_*$ and the standard deviation values, σ, are taken from the predicted covariance function with the added observation noise, or $\mathrm{cov}(\mathbf{f}_*) + \sigma_n^2 I$.

The Matern kernel is used for the covariance function in this work[5]. The general form for the Matern class of covariance functions is given by,

$$k_{\text{Matern}}(r) = \frac{2^{1-\nu}}{\Gamma(\nu)}\left(\frac{\sqrt{2\nu}r}{l_k}\right)^{\nu} K_{\nu}\left(\frac{\sqrt{2\nu}r}{l_k}\right), \qquad \text{S5}$$

where $r = |\mathbf{x} - \mathbf{x}'|$ is the Euclidean distance between two points in the input parameter space, ν is a smoothing parameter, l_k is a length-scale parameter, Γ is the gamma function, and K_ν is a modified Bessel function. The smoothing parameter ν is typically selected, not learned, such that the function assumes the convenient form of the product of a polynomial and exponential. This work uses the Matern kernel with $\nu = 5/2$, which has the following form,

$$k_{\nu=5/2}(r = |\mathbf{x} - \mathbf{x}'|) = \sigma_k\left(1 + \frac{\sqrt{5}r}{l_k} + \frac{5r^2}{3l_k^2}\right)\exp\left(-\frac{\sqrt{5}r}{l_k}\right), \qquad \text{S6}$$

where σ_k is the scale factor parameter. The scale factor controls the variance of the distribution at a given location. The length scale controls the rate at which the functions vary between two $\mathbf{x}$ locations, where smaller length scales give more rapidly varying functions while larger length scales give smoother functions.

The model used in this work is a multi-task (multi-output) GP regression model that is based on the work of Bonilla et al.[7]. This expands the covariance function of the GP model to include hyperparameters that allow for learning between each of the tasks', or outputs', covariance functions. This can allow for more accurate predictions with less data when the trends for each of the outputs are sufficiently correlated. With this method the covariance function between two points $\mathbf{x}$ and $\mathbf{x}'$ for two different tasks *i* and *j* now become

$$k([\mathbf{x}, i], [\mathbf{x}', j]) = k_{XX}(\mathbf{x}, \mathbf{x}') \otimes k_{TT}(i, j) \qquad \text{S7}$$

where $\otimes$ is a Kronecker product, k_{XX} is the typical covariance function, like in Eq. S6, and k_{TT} is a simple inter-task covariance matrix of weights that specifies the inter-task similarities. Using

this new covariance function the standard method of inference in Eqs. S1-S4 can be used where the covariance matrix K is now computed using Eq. S7.

While GP regression provides a closed-form solution for predictions based on a distribution conditioned on a set of training data, the accuracy of the model can be further improved through standard learning methods for the model's hyperparameters. Typical loss functions used in machine learning, such as mean squared error, do not allow for consideration of the probabilistic properties of a GP. To do this, a new loss function can be used, the log marginal likelihood[5]. The log marginal likelihood of a GP model is differentiable and can be maximized using the numerical methods common to machine learning, such as Adam optimization[8]. The marginal likelihood is the likelihood times the prior[5],

$$p(\mathbf{y}|X) = \int p(\mathbf{y}|\mathbf{f}, X)\, p(\mathbf{f}|X)\, d\mathbf{f} \qquad \text{S8}$$

where the $\mathbf{y}$ is the vector of likelihoods for X, and $\mathbf{f}$ is the function values of the prior distribution for X. Since our training data has already been observed, we know a good GP model should predict a high probability of $\mathbf{y}$ given X. Hence, why the marginal likelihood should be maximized. It is convenient to work with the log of the marginal likelihood, and we can integrate Eq. S8 to arrive at the log marginal likelihood[5],

$$\log p(\mathbf{y}|X) = -\frac{1}{2}\mathbf{y}^{\mathrm{T}}\left(K + \sigma_n^2 I\right)^{-1}\mathbf{y} - \frac{1}{2}\log\left|K + \sigma_n^2 I\right| - \frac{n}{2}\log 2\pi\,, \qquad \text{S9}$$

where K is the covariance matrix for $K(X, X)$, and n is the number of training data points. The log marginal likelihood is maximized in the GP training process. Overfitting of the training dataset is avoided by selecting the hyperparameters from the training iteration which yields the maximum log marginal likelihood for the validation dataset.

Once we have a sufficient set of learned hyperparameters, we can use the GP model to predict the mean and variance of the output parameters at a given location in the input parameter space. Using Eqs. 1 and 2 in the main text we can obtain a mean, μ_{MSE}, and standard deviation, σ_{MSE}, to use in our Expected improvement (EI) acquisition function. A more in-depth examination of the EI function and other common acquisition functions can be found in Garnett[9]. The EI function evaluates new locations in the parameter space and compares them to the best observed experimental data point to determine their *expected improvement* on the existing best-observed point. Let μ^*_{MSE} be the minimum shape error value for the experimentally obtained dataset. Using this value, we can create a utility function: $u(\mathbf{x}) = \max\left(0, \mu^*_{MSE} - \mu_{MSE}(\mathbf{x})\right)$ that evaluates the improvement at a given location, only selecting the difference if the new location improves on the previous best point, otherwise selecting zero. Then EI is the expectation of improvement of this utility function under the distribution given by our model and training data[9].

$$\mathrm{EI}(\mathbf{x}) = \mathbb{E}\left[u(\mathbf{x}) \middle| \mathbf{x}, X, \mathbf{y}\right] = \int_{-\infty}^{\mu^*} \left(\mu^* - \mu(\mathbf{x})\right) \mathcal{N}\left(\mu(\mathbf{x}), \sigma(\mathbf{x})\right) d\mu \qquad \text{S10}$$

where we have dropped the MSE subscript for compactness and $\mathcal{N}\left(\mu(\mathbf{x}), \sigma(\mathbf{x})\right)$ is the distribution predicted by the GP model. This expectation can be evaluated in closed form, arriving at[9],

$$\mathrm{EI}(\mathbf{x}) = \left(\mu^* - \mu(\mathbf{x})\right) \Phi\left(\frac{\mu^* - \mu(\mathbf{x})}{\sigma(\mathbf{x})}\right) + \sigma(\mathbf{x}) \phi\left(\frac{\mu^* - \mu(\mathbf{x})}{\sigma(\mathbf{x})}\right), \qquad \text{S11}$$

where Φ and ϕ are the cumulative distribution function and probability distribution function of the standard normal distribution, respectively. This equation for EI returns a scalar value and can be maximized with a standard numerical method to determine a set of input parameters $\mathbf{x}$ that are most likely to improve the model for determining the minimal shape error. In this work, given the

complexity of our multi-dimensional input and output spaces, we use Powell's conjugate direction method[10] for optimization due to its computational efficiency and gradient-free methodology.

Supplementary Note 6: Detailed Results for BO Framework Test Cases

The qualitative and quantitative results for all nine test cases are shown here. Figures S8-S10 are the qualitative results for circle, square, and triangle shapes, respectively. Each figure contains the results for each size. The sizes are ordered by row with 27-µm on top, 13.5-µm in the middle, and 6.75-µm on the bottom. From left to right the images are the target pattern, the printed result of the target pattern compared with the target shape, the optimal pattern determined by the framework, the grayscale image of the printed structure, the binarized image of the printed structure, the grayscale image overlayed with the detected binary shape, and the binary shape compared with the target shape.

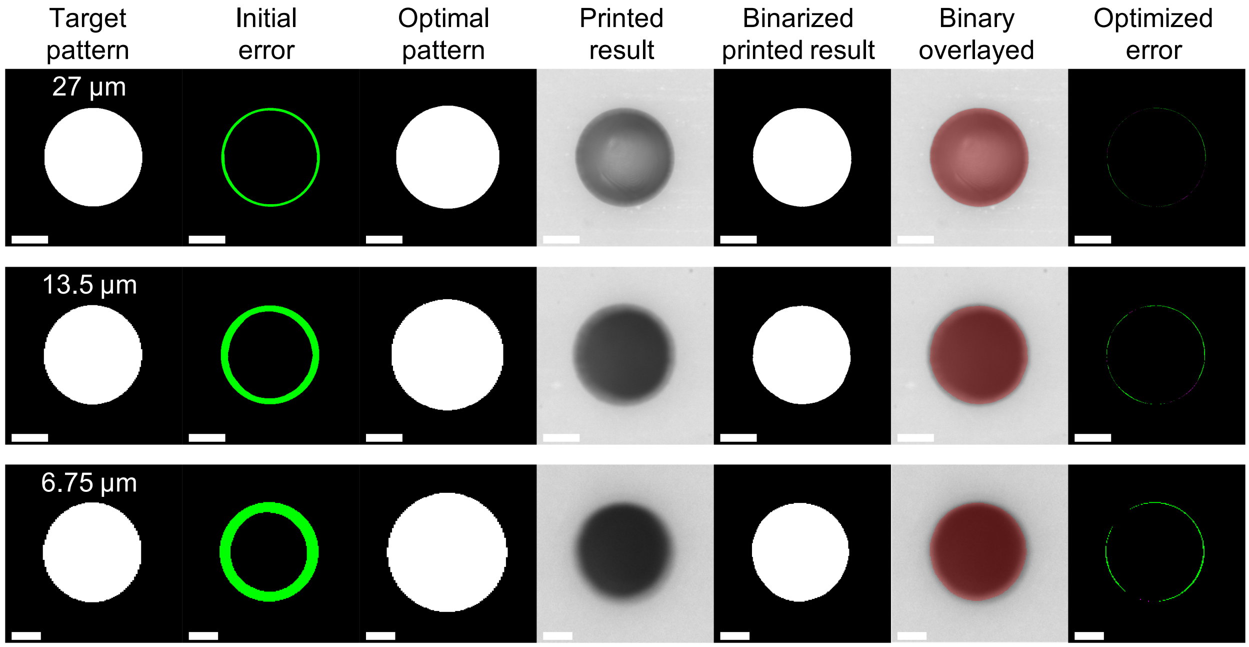

Fig. S8 Qualitative results for circle shapes. The sizes are ordered by row with the 27-µm results on the top row, 13.5-µm results in the middle, and 6.75-µm

results on the bottom row. Column 1 shows the target pattern. Column 2 presents the printed result of the target pattern compared with the target shape. Column 3 illustrates the optimal pattern determined by the framework. Column 4 introduces the printed result of the optimal pattern. Column 5 reveals the binary shape detected in the grayscale image. Column 6 demonstrates the binary shape overlayed on the grayscale image. Column 7 indicates the printed results of the optimal pattern compared with the target shape. The scale bars for the top row are 10 μm, while the scale bars for the middle row are 5 μm, and the bottom row are 2 μm.

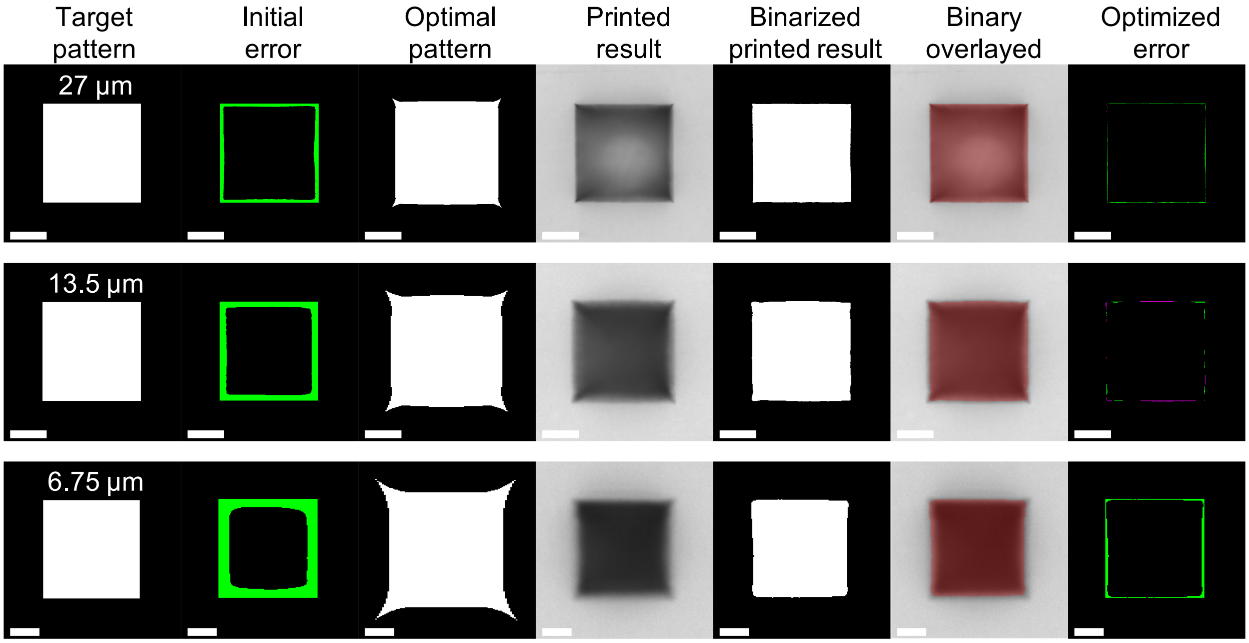

Fig. S9 Qualitative results for square shapes. The sizes are ordered by row with the 27-μm results on the top row, 13.5-μm results in the middle, and 6.75-μm results on the bottom row. Column 1 shows the target pattern. Column 2 presents the printed result of the target pattern compared with the target shape. Column 3 indicates the optimal pattern determined by the framework. Column 4 reveals the printed result of the optimal pattern. Column 5 displays the binary shape detected

in the grayscale image. Column 6 demonstrates the binary shape overlayed on the grayscale image. Column 7 introduces the printed results of the optimal pattern compared with the target shape. The scale bars for the top row are 10 µm, the middle row are 5 µm, and the bottom row are 2 µm.

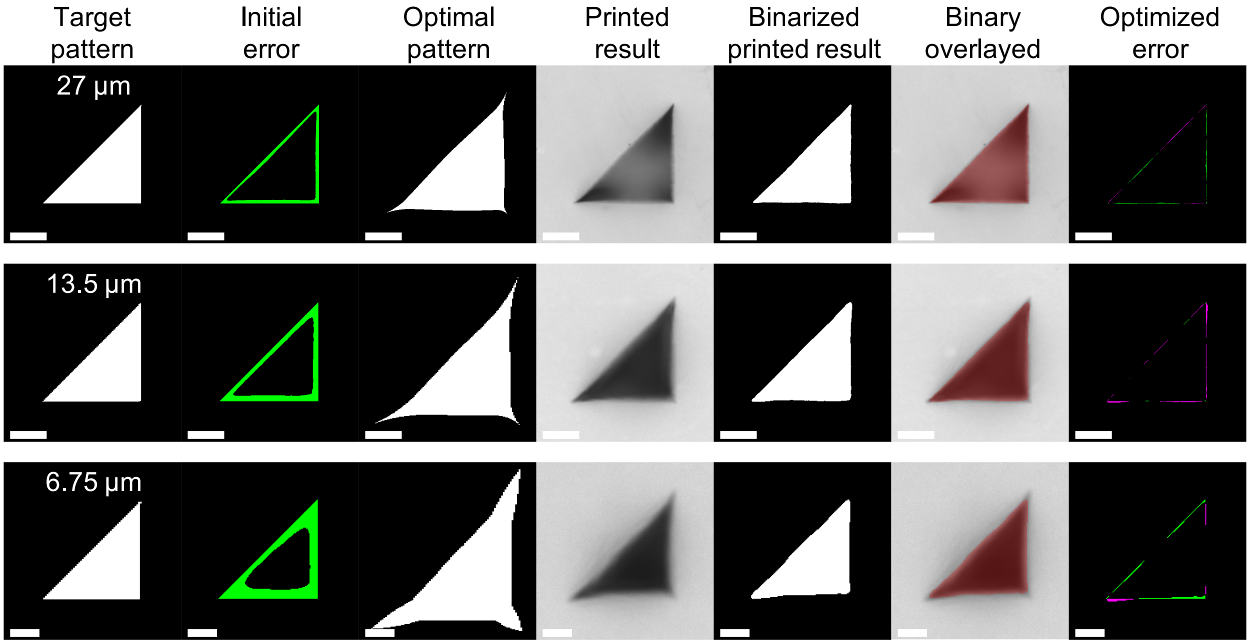

Fig. S10 Qualitative results for triangle shapes. The sizes are ordered by row with the 27-µm results on the top row, 13.5-µm results in the middle, and 6.75-µm results on the bottom row. Column 1 shows the target pattern. Column 2 presents the printed result of the target pattern compared with the target shape. Column 3 indicates the optimal pattern determined by the framework. Column 4 reveals the printed result of the optimal pattern. Column 5 introduces the binary shape detected in the grayscale image. Column 6 displays the binary shape overlayed on the grayscale image. Column 7 demonstrates the printed results of the optimal pattern compared with the target shape. The scale bars for the top row are 10 µm, the middle row are 5 µm, and the bottom row are 2 µm.

The quantitative results from each BO iteration for each shape are shown in Figs. S11-S13 for 27-µm, 13.5-µm, and 6.75-µm shapes, respectively. Each figure contains the results for each of the five outputs, arranged in rows, with the circle results in the first column, the square results in the second column, and the triangle results in the third column.

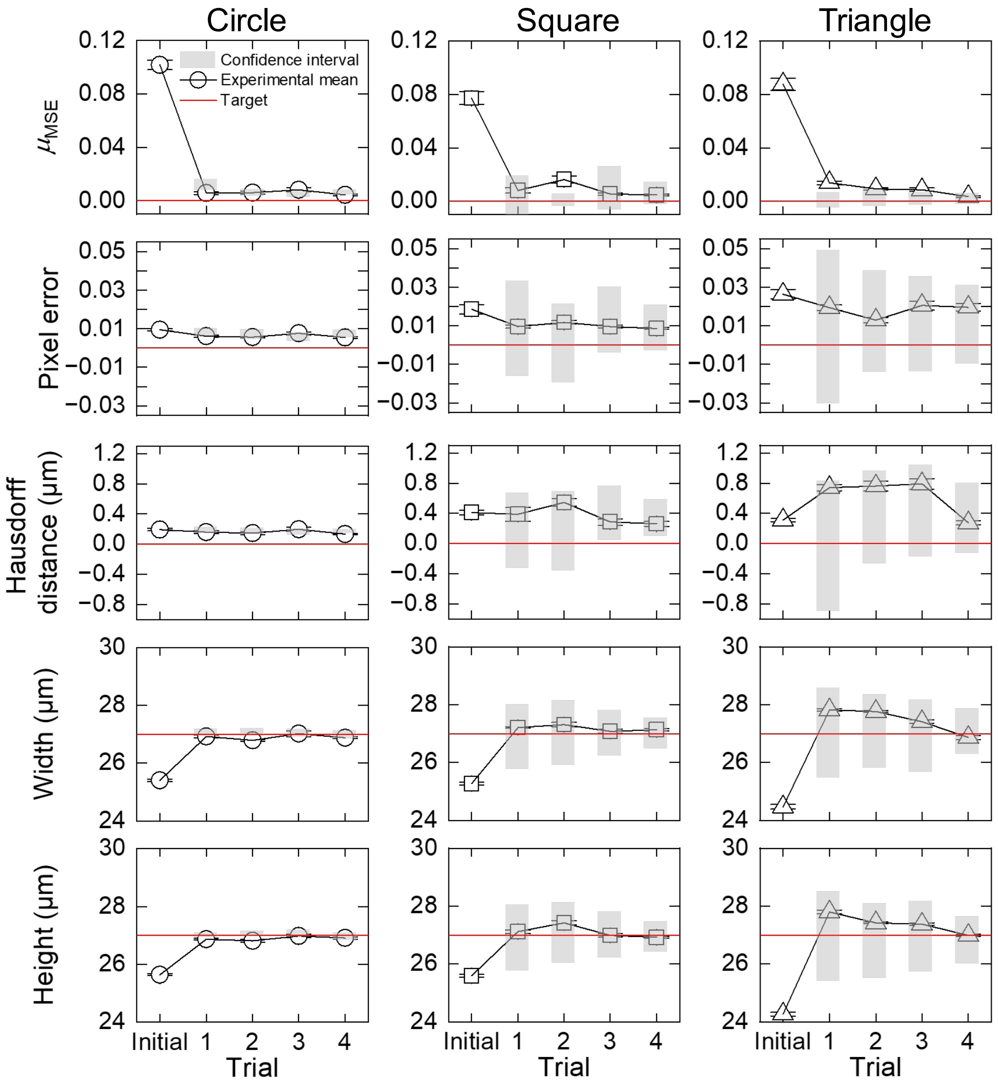

Fig. S11 Quantitative results for 27-µm shapes. Experimental results for each output parameter per iteration of Bayesian optimization. The error bars indicate the experimental ± one standard deviation range. The gray bar is the ± one standard deviation confidence interval predicted by the GP regression model. The red line is

the target value. The first column is the circle results. The second column is the square results. The third column is the triangle results.

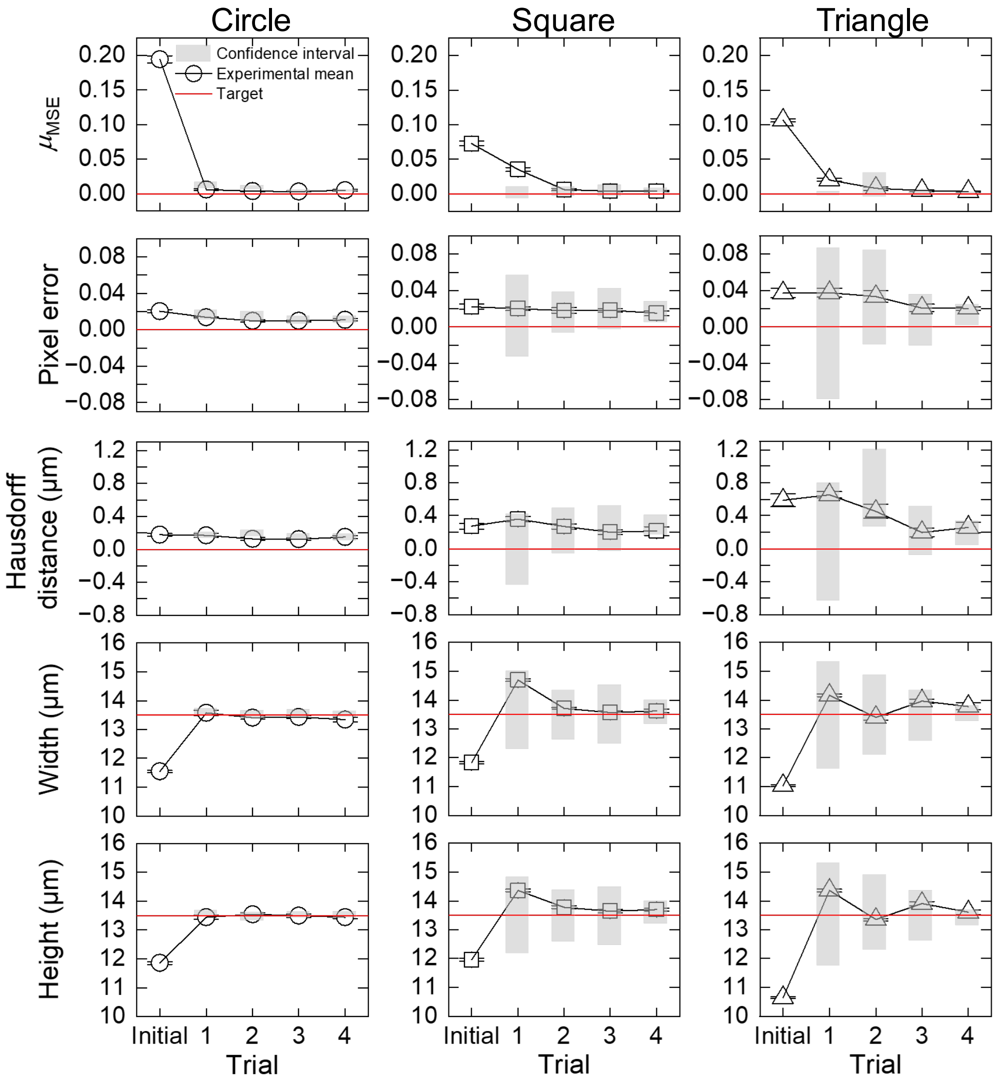

Fig. S12 Quantitative results for 13.5-μm shapes. Experimental results for each output parameter per iteration of Bayesian optimization. The error bars indicate the experimental ± one standard deviation range. The gray bar is the ± one standard deviation confidence interval predicted by the GP regression model. The red line is the target value. The first column is the circle results. The second column is the square results. The third column is the triangle results.

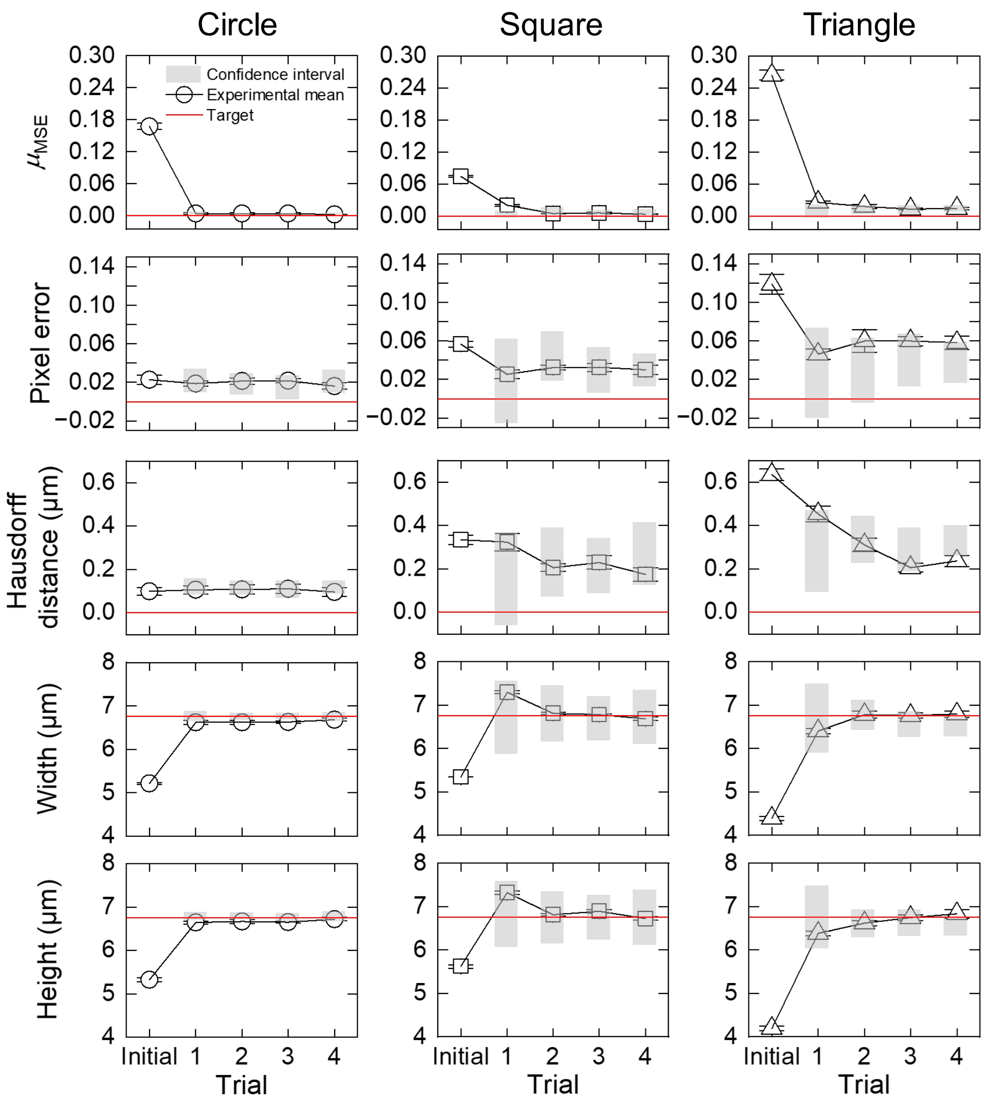

Fig. S13 Quantitative results for 6.75-µm shapes. Experimental results for each output parameter per iteration of Bayesian optimization. The error bars indicate the experimental ± one standard deviation range. The gray bar is the ± one standard deviation confidence interval predicted by the GP regression model. The red line is the target value. The first column presents the circle results. The second column illustrates the square results. The third column introduces the triangle results.

Supplementary Note 7: Bayesian Optimization of Other Shapes

The BO framework was used to optimize two additional polygons of greater complexity, a 6.75 µm pentagon and a 6.75 µm 5-pointed star. These results were printed at a laser power of 540 mW and exposure time of 2 ms instead of the 340 mW and 10 ms exposure time used for the other shapes to test the framework's ability to optimize for other laser parameters. The qualitative results for these shapes are shown in Fig. S14. The quantitative results are shown in Fig. S15. The perimeter error for the pentagon was reduced from an initial value 0.539 µm to an optimized value of 0.049 µm in four trials of the BO framework. The perimeter error for the star was reduced from an initial value of 0.414 µm to an optimized value of 0.064 µm in three trials of the BO framework.

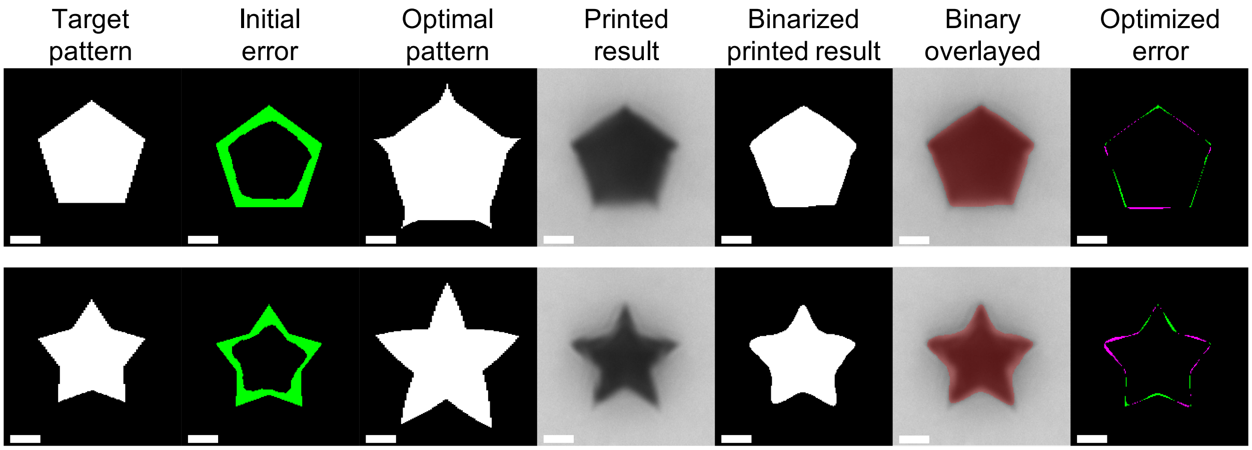

Fig. S14 Qualitative results for 6.75-µm pentagon and star shapes. The results for the pentagon are shown in the top row, and the results for the star are shown in the bottom row. Column 1 shows the target pattern. Column 2 presents the printed result of the target pattern compared with the target shape. Column 3 indicates the optimal pattern determined by the framework. Column 4 reveals the printed result of the optimal pattern. Column 5 introduces the binary shape detected in the grayscale image. Column 6 displays the binary shape overlayed on the grayscale

image. Column 7 demonstrates the printed results of the optimal pattern compared with the target shape. The scale bars in all images are 2 μm.

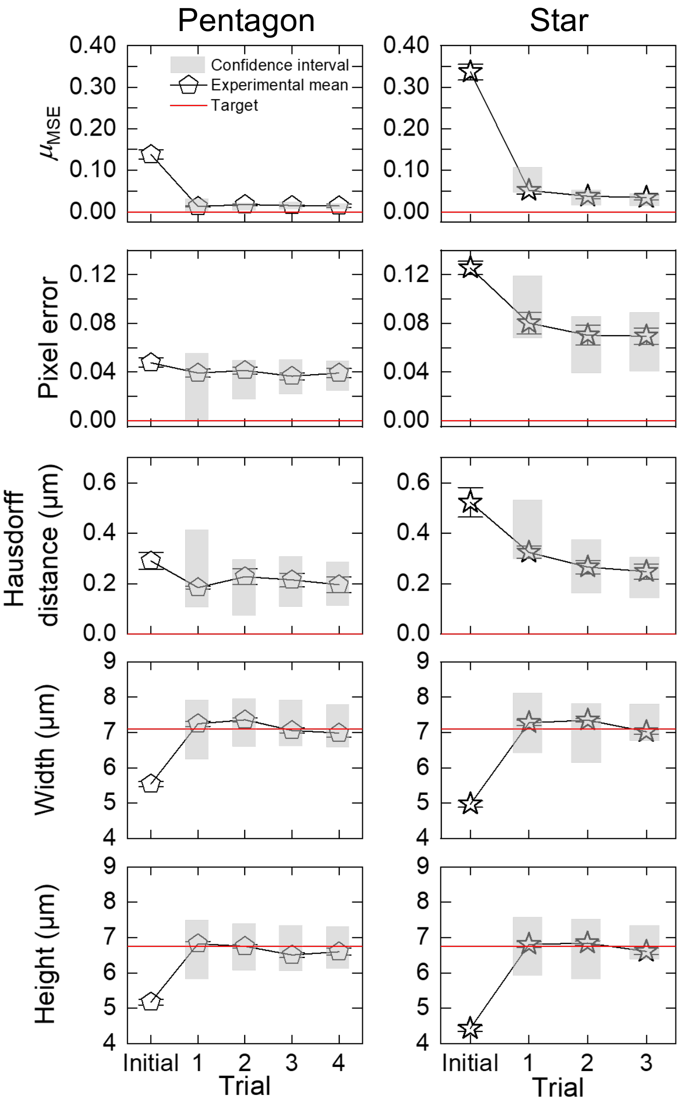

Fig. S15 Quantitative results for 6.75-μm pentagon and star shapes. Experimental results for each output parameter per iteration of Bayesian optimization. The error bars indicate the experimental ± one standard deviation range. The gray bar is the ± one standard deviation confidence interval predicted by the GP regression model. The red line is the target value. The first column presents the pentagon results. The second column presents the star results.

Supplementary Note 8: Comparison of Convolutional Neural Network with BO Framework

To further evaluate the performance of our BO framework, we have trained a convolutional neural network (CNN) on the BO framework's nine base datasets for comparison. CNNs that output images based on an input image are typically called autoencoders. One common autoencoder for image analysis is U-Net[11], which was first introduced for biomedical image segmentation. After implementing the U-Net structure in PyTorch we observed high computational cost and poor performance and accuracy of the output images. AlexNet[12] is a CNN first presented for image classification and is particularly efficient for training in GPU-enabled environments. Here, we present an enhanced AlexNet autoencoder structure, which outperforms the U-Net structure in both training time and prediction accuracy. Figure S16 shows this structure, where the original AlexNet structure is mirrored over its output node with skip connections branching between each of the encoder/decoder layers. This CNN was implemented using PyTorch. Many practical uses for CNN models benefit from inverse prediction. In this case, to optimize DMD patterns an inverse design was trained which predicts the input patterns necessary to generate a desired target shape.

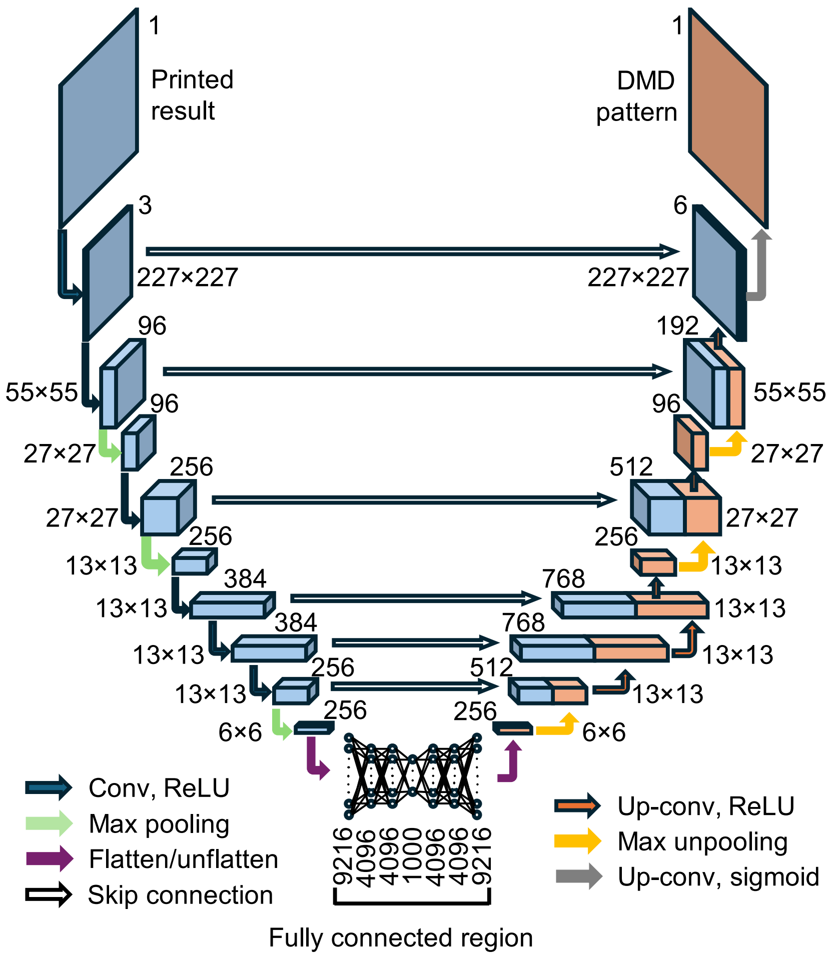

Fig. S16 AlexNet autoencoder structure. The CNN structure used for comparison in this work is based off the AlexNet[12] CNN structure. To transform the CNN into an autoencoder structure AlexNet is mirrored over the fully connected region. The CNN structure is an inverse model, taking the printed result as an input and outputting the DMD pattern necessary to produce that result.

For comparison with the BO framework, the CNN model was trained on the BO framework base datasets for all three shapes and all three sizes. This yields a dataset of 3067 input and output images which are split 80/20 for training/validation. The binarized images of printed results are used as the inputs to train the CNN to predict the corresponding DMD patterns as outputs. Table S2 shows the perimeter error results for the BO framework and for the DMD patterns predicted by

the trained CNN model for 6.75 μm target shapes. The error results for the CNN are 2-5× larger than the BO framework despite having 4-5 times more training data points. Figure S17 illustrates the qualitative results for the CNN. Compared to the results obtained from BO shown in Fig. 4 of the main text, it is evident that the CNN predictions yield printed results with larger errors.

Table S2. BO and CNN results for perimeter error of 6.75-μm shapes.

Shape	Initial (μm)	BO (μm)	CNN (μm)
Circle	0.647	0.051	0.299
Square	0.627	0.116	0.276
Triangle	0.494	0.054	0.156

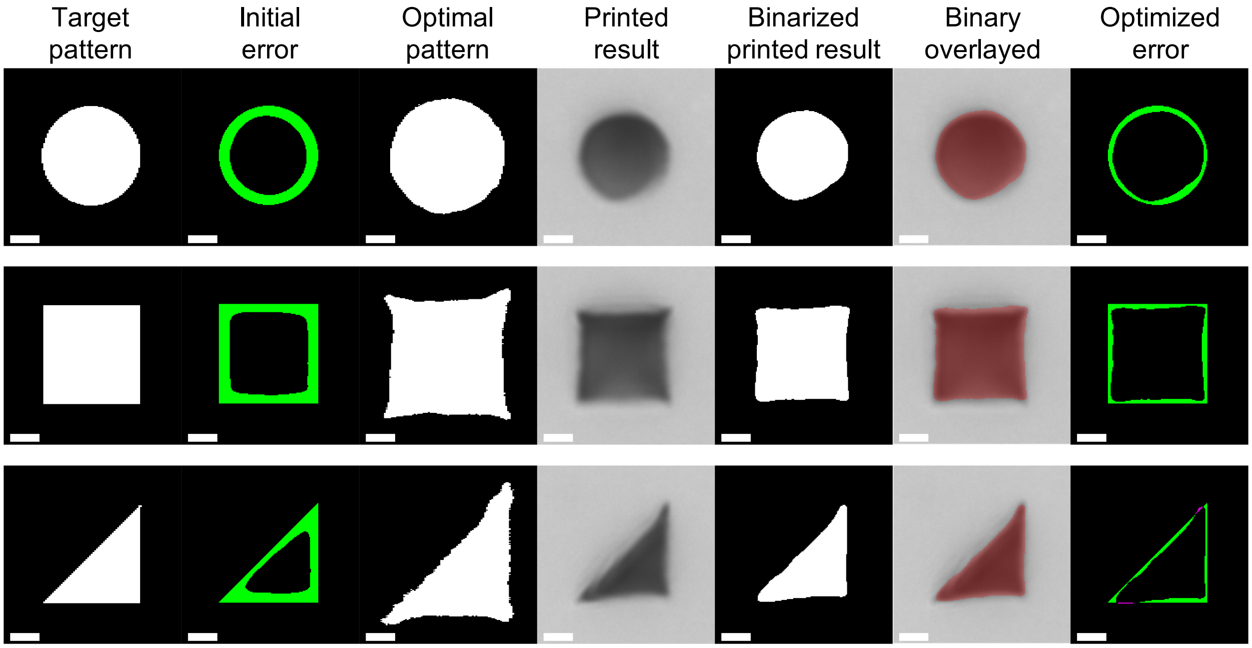

Fig. S17 Printed results for CNN prediction of DMD patterns for 6.75-μm target shapes. Column 1 shows the target pattern. Column 2 presents the printed result of the target pattern compared with the target shape. Column 3 indicates the optimal pattern predicted by the CNN. Column 4 reveals the printed result of the optimal pattern. Column 5 shows the binary shape detected in the grayscale image. Column 6 displays the binary shape overlayed on the grayscale image. Column 7

shows the printed results of the optimal pattern compared with the target shape. The scale bars in all images are 2 μm.

Supplementary References

1. J, M. Find vertices in image of convex polygon. *MATLAB Central File Exchange* Preprint at https://www.mathworks.com/matlabcentral/fileexchange/74181-find-vertices-in-image-of-convex-polygon (2024).
2. You, S. *et al.* Mitigating Scattering Effects in Light-Based Three-Dimensional Printing Using Machine Learning. *J Manuf Sci Eng* **142**, (2020).
3. Zhou, C., Xu, H. & Chen, Y. Spatiotemporal Projection-Based Additive Manufacturing: A Data-Driven Image Planning Method for Subpixel Shifting in a Split Second. *Advanced Intelligent Systems* **3**, 2100079 (2021).
4. Somers, P. *et al.* Rapid, continuous projection multi-photon 3D printing enabled by spatiotemporal focusing of femtosecond pulses. *Light Sci Appl* **10**, 199 (2021).
5. Rasmussen, C. E. & Williams, C. K. I. *Gaussian Processes for Machine Learning*. (The MIT Press, 2005). doi:10.7551/mitpress/3206.001.0001.
6. Gardner, J., Pleiss, G., Weinberger, K. Q., Bindel, D. & Wilson, A. G. GPyTorch: Blackbox Matrix-Matrix Gaussian Process Inference with GPU Acceleration. in *Advances in Neural Information Processing Systems* (eds. Bengio, S. et al.) vol. 31 (Curran Associates, Inc., 2018).
7. Bonilla, E. V, Chai, K. & Williams, C. Multi-task Gaussian Process Prediction. in *Advances in Neural Information Processing Systems* (eds. Platt, J., Koller, D., Singer, Y. & Roweis, S.) vol. 20 (Curran Associates, Inc., 2007).

8. Kingma, D. P. & Ba, J. Adam: A Method for Stochastic Optimization. *International Conference on Learning Representations (ICLR)* (2015).

9. Garnett, R. *Bayesian Optimization*. (Cambridge University Press, Cambridge, 2023). doi:10.1017/9781108348973.

10. Powell, M. J. D. An efficient method for finding the minimum of a function of several variables without calculating derivatives. *Comput J* **7**, 155–162 (1964).

11. Ronneberger, O., Fischer, P. & Brox, T. U-Net: Convolutional Networks for Biomedical Image Segmentation. in *Medical Image Computing and Computer-Assisted Intervention – MICCAI 2015* (eds. Navab, N., Hornegger, J., Wells, W. M. & Frangi, A. F.) 234–241 (Springer International Publishing, Cham, 2015).

12. Krizhevsky, A., Sutskever, I. & Hinton, G. E. ImageNet Classification with Deep Convolutional Neural Networks. in *Advances in Neural Information Processing Systems* (eds. Pereira, F., Burges, C. J., Bottou, L. & Weinberger, K. Q.) vol. 25 (Curran Associates, Inc., 2012).
